# “Surthriving” Hand Rehabilitation: Proposing Interventions to Support Novice Occupational Therapists Working in Underserved Contexts

**DOI:** 10.1155/2023/5562025

**Published:** 2023-12-14

**Authors:** Kirsty van Stormbroek, Tania van der Merwe, Lisa O'Brien, Hellen Myezwa

**Affiliations:** ^1^Department of Occupational Therapy, School of Therapeutic Sciences, University of the Witwatersrand, Johannesburg, South Africa; ^2^Swinburne University of Technology Hawthorn Campus, John Street, Hawthorn, Australia; ^3^Department of Physiotherapy, School of Therapeutic Sciences, Faculty of Health Sciences, University of the Witwatersrand, Johannesburg, South Africa

## Abstract

Hand rehabilitation delivered to underserved South African communities is often the responsibility of novice or generalist occupational therapists. Novice therapists typically work with restricted supervision, support, and resources. Little is known about how these therapists should be supported in order to strengthen the services that they deliver. This study is aimed at understanding how novice occupational therapists in their first year of practice describe their experience of delivering hand rehabilitation in order to identify their support and development needs and propose interventions to address these needs. A qualitative instrumental case study design was used. Data were collected from novice occupational therapists (*n* = 9) who participated in an online community of practice. Data collection techniques included photoelicitation, facilitated reflection activities, and case discussion. Reflexive thematic analysis was employed. Trustworthiness strategies included reflexive writing, prolonged engagement, data source triangulation, member checking, and peer audit. Analysis generated three themes: (1) “submerged: I had to drown a little” captured participants' experience of being saturated by contextual features including poverty and poor basic management of hand injuries. (2) “Starting somewhere” captured participants' journey of treating patients with hand injuries. They transitioned from an initial sense of having “no idea” to developing “some idea”; their clinical reasoning was challenged when working with no diagnosis, unfamiliar presentations, or when contextual features rendered traditional approaches to therapy inappropriate. Finally, (3) “dynamics of ‘surthrival'” captured elements that contributed to participants either thriving or merely surviving their hand rehabilitation experience. The proposed strategies identified in this study to address the support and development needs of novice therapists include interventions focused on systems and health services; learning opportunities to support competency and physical resources; and emotional support. Beyond application to the South African context, these strategies may be considered for supporting generalist or novice therapists delivering hand rehabilitation in other low- to middle-income countries.

## 1. Introduction

Hand injuries are common. Estimated to account for 20% of injuries presenting at hospital emergency departments [1], 34.4 million emergency department encounters were reported in the United States between 2009 and 2012, representing $180,4 billion in health care charges [2]. In two high-income countries, 29% of all unintentional injuries were hand injuries [3]. The burden of hand injuries in low- to middle-income countries (LMIC) is difficult to quantify due to gaps in hand injury data; however, this burden has been described as substantial and increasing [4, 5]. Despite limited data, sufficient evidence exists for hand injuries in LMICs to be considered a public health priority [4, 6, 7].

South Africa is no exception. Around 17 000 hand-injured patients are treated each year at just one of the country's public hand surgery units [8]. In contrast to many other countries, interpersonal violence is central to the burden of hand injuries in South Africa [8, 9] and is commonly related to alcohol misuse [10–12]. Occupational injuries and road accidents also contribute to the burden [9, 13, 14], as is common in other LMICs [15–17]. Resources to respond to the burden of hand injuries vary considerably: the availability and quality of healthcare is uneven across South Africa with the majority of healthcare resources servicing the minority of citizens that have access to private healthcare [18]. Evidence suggests the same trend for hand-injury care services: hand care expertise is concentrated in the country's private sector and urban provinces [19], and most hand conditions are treated by health professionals insufficiently trained in hand injury management [20]. High rehabilitation staff turnover in the public sector [21] and the severe shortage of occupational therapists in South Africa (1: 10 000 population) [22] compound these challenges. Accessing scarce services is complicated by high levels of poverty and unemployment (55.5% [23] and 42.4% [24] of the population, respectively).

Rehabilitation after hand injury is essential. Most hand injuries are sustained by adults of an economically active age. Rehabilitation for individuals across the lifespan is important, but even more so for those whose injury to “the earning tool” [25] may devastate their livelihood. Rehabilitation of the hand is an area of occupational and physiotherapy practice that is commonly considered to require specialized skills [26, 27]. Despite this, this service very often falls to novice or generalist occupational therapists in the public health sector of South Africa. These therapists are either fully registered occupational therapists working in a generalist capacity or new graduates completing a year of compulsory community service (CS) in rural or underserved areas directly after graduation. They are required to deliver services across areas of occupational therapy practice, including hand rehabilitation.

The experience of community service occupational therapists (CSOTs) delivering hand-injury care was the focus of this study. Despite being in their first year of practice, these therapists work as generalists, have limited experience, and commonly receive poor support, supervision, and resources for their work [28, 29]. They are also often responsible for delivering occupational therapy services to rural populations who are considered to have the greatest healthcare needs in a context that is often complex [30, 31] and requires experience to navigate. Knowledge regarding their experiences and the needs and demands placed on these therapists for delivering hand rehabilitation was captured in one previous quantitative study [29]. However, an understanding of their support and development needs for hand rehabilitation, and how these needs should be met in a contextually responsive manner, remains unexplored.

This study thus sought to:

- Describe how CSOTs experience their delivery of hand rehabilitation
- Describe the support and development needs that emanate from this experience
- Identify strategies and interventions to strengthen the capacity of these therapists to deliver essential hand rehabilitation services

## 2. Materials and Methods

A qualitative descriptive instrumental multiple-case study design [32] was employed. This design recognizes that a case and context cannot be easily separated [32], which is particularly relevant for the population being studied as contextual features are known to influence their practice [28]. The case study was instrumental to enable a study of therapists' experience to produce an understanding of their support and development needs. The design resonated with the constructivist beliefs that directed the inquiry towards understanding the meaning that participants ascribed to their hand rehabilitation experience. Further details on the first author's positionality may be viewed in Supplementary File 1.

Purposive sampling is recommended for case selection in case study research [33]. However, it was not possible to predict which CSOTs in a given year routinely treat patients with, or at risk of hand injuries. Therefore, invitations to participate in the study were distributed to CSOTs via professional organisations and social media. CSOTs who communicated interest in the study were invited to an online information session. Informed consent was obtained from nine CSOTs who were added to the online community of practice (CoP). Each participant assigned themselves a very specific pseudonym for anonymity and also to portray themselves within their CSOT role. Participants were spread across five of the nine South African provinces, in both rural (*n* = 5) and urban (*n* = 4) locations. Three participants (*n* = 3) were employed at primary healthcare clinics (the first step in facility-based healthcare services), three participants (*n* = 3) at district hospitals (district hospitals receive referrals from and provide support to primary healthcare clinics and community health centres), and three participants (*n* = 3) at regional hospitals (regional hospitals provide specialist support to district hospitals) [34]. A basic profile of each participant is outlined in Table 1. Ethical clearance was obtained from the The University of the Witwatersrand Human Research Ethics Committee (Medical) (M200235).

Data collection occurred between July 2021 and January 2022. The CoP met fortnightly on MS Teams and interacted on WhatsApp. Audio recordings of the meetings were transcribed, and the WhatsApp group chat was downloaded. Data collection techniques used within meetings included photo elicitation [35] and facilitated reflection activities. Professional development activities were planned in response to emerging needs. Transcriptions of the MS Teams meetings as well as the downloaded WhatsApp group chat, along with photos, illustrations, and facilitated reflection activities, were uploaded to NVivo 12 for thematic analysis. Further detail of data collection activities is presented in Table 2.

Braun and Clarke's reflexive thematic analysis (RTA) was used [33, 38]. The iterative steps of RTA taken were as follows: (1) data familiarisation (listening to recordings, reading transcriptions, and making notes); (2) generating codes (all data coded inductively with some deductive codes added; a finalised codebook applied across all data); (3) constructing themes (codes and associated data examined and meaningful data patterns constructed through collapsing, combining, and clustering codes [39]); (4) reviewing potential themes with the study's participants and coauthors; (5) defining and naming the themes, and finally, (6) constructing the report. In keeping with the case study design, a vignette was constructed as a means of representing the data [40]. Due to the collaborative nature of data collection and a focus on cross-case analysis in this study, a composite vignette was chosen to provide a single account representative of all participants' experiences [40]. To compose the vignette, the first author reviewed participants' written reflections from the sentence stem reflective activity (see Table 2). Phrases that aptly captured contextual detail or key aspects of participants' experiences were highlighted for use in the vignette. The author's prolonged engagement with participants and data enabled her to weave imagery from photos and transcribed data into the depiction of participants' contexts and experiences captured in the vignette. Each element of the vignette can be traced back to the data. The vignette was also presented to members on two occasions to check that they could “recognise their experience in the research findings” (p.219) [41] and ensure that the vignette captured an authentic representation of their collective experience.

In order to propose strategies and interventions for therapists' support and development, the researcher systematically appraised each theme and subtheme. Notes were made of the experiences, needs, and demands described in each, and strategies and interventions were conceptualised to address these. The researcher reviewed the notes of proposed strategies and sorted these according to similar types or levels of intervention. The four categories were named as follows: systems and services; learning opportunities to support competence; physical resources; and emotional support. The proposed list of strategies within each category was presented to and discussed with participants in two online member-checking meetings. Participants provided verbal feedback on the strategies with some additional ideas being added while some participants chose to record their responses (e.g., participant 9) on an Excel document. Strong agreement was recorded, and additional ideas were added to the list of interventions (see Supplementary File II).

Reflexive thematic analysis, as an approach to thematic analysis, harnesses the researcher's subjectivity rather than seeking to attain a contrived sense of objectivity in analysis. The first author's personal experience as a CSOT in 2006 and prior research with the same population enabled her to ask insightful questions and probe astutely. However, RTA also directed her to evaluate and articulate her assumptions, perceptions, and positionality, and the potential impact of her subjectivity on the research process [42]. The use of reflexivity contributed to the strategies employed to ensure research rigor.

Rigor was pursued through attending to four components of trustworthiness [41]. Credibility was enabled through data source triangulation and reflexivity. The latter was pursued through reading [42, 43] and self-critical writing in which assumptions and positionality were examined (see Supplementary File 1). The first author examined the impact that a previous undergraduate lecturer-student relationship with five of the participants could have on power dynamics, as well as the freedom with which these participants shared. Conversely, the impact of these participants' familiarity with the researcher on the other four participants was considered, as well as the efforts made to support the sharing and engagement of all participants. The first author examined the impact that her own community service experience had on her interpretation of the data addressing the risk of more readily identifying and probing experiences that resonated with her own [39]. Prolonged engagement was enabled by frequent online interaction with participants from July 2021 to September 2022. In addition, robust member checking was conducted within the data collection/early analysis process, as well as after the analysis. Participants were presented with the research findings in an online group meeting and follow-up communication with individual participants. Strong agreement with the vignette, themes, and subthemes was recorded (see Supplementary File II). Participants recommended changes to the names of two subthemes to capture the range of experiences more accurately within the group. Participants also qualified their agreement with each subtheme to ensure that variance was appropriately captured in reporting. Data source triangulation and reflexive actions enabled confirmability of findings, while a description of participants' practice settings enable readers to evaluate the transferability of the findings. Dependability of findings was established through a dense description of research methods exposed to frequent scrutiny by the study's coauthors and by undergoing a formal auditing process by an experienced research colleague at the completion of analysis. The report submitted for auditing, which includes the finalised codebook, may be viewed here (Supplementary file III). Data is available on reasonable request from the first author.

## 3. Results

Results generated from the analysis are presented in three parts: a composite vignette of participants' experiences is presented in Table 3 followed by the themes and subthemes constructed from analysis. The proposed interventions to address the support and development needs identified in the data are presented at the end of this section.

**Figure 1:**
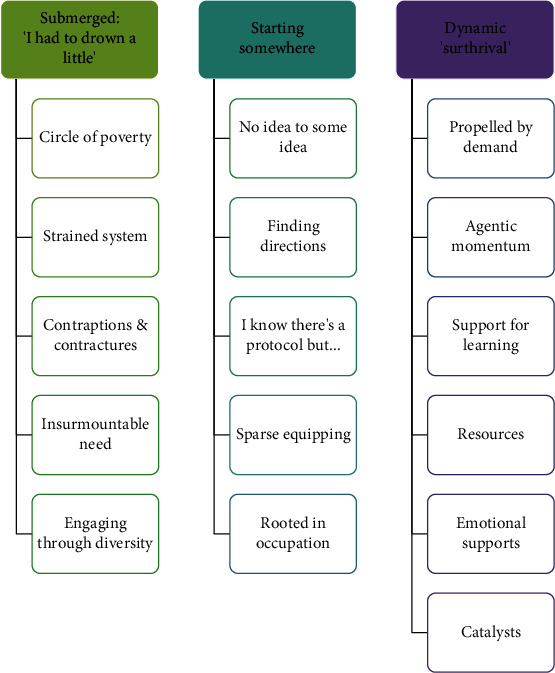
Themes and subthemes constructed through reflexive thematic analysis.

Three themes and a number of subthemes were generated from the analysis. These are illustrated in Figure 1, and an account of each theme is provided thereafter.

### 3.1. Theme 1: Submerged—“I Had to Drown a Little”

The name of theme 1 was drawn from the imagery suggested by one of the participants who said, “I had to drown a little” (Illusion-less Optimist). The theme describes the contextual features that characterised participants' experiences.

#### 3.1.1. Circle of Poverty

The first subtheme, circle of poverty, captures the pervasive poverty that characterised patients' lives and encircled participants' work:

“People's lives are characterised by…their low socioeconomic statuses. Their livelihood(s) are focused on survival. Their occupations… are unfulfilled.” Bad Grad

Echoing this description, numerous references to poverty were made by all participants. One of the rural therapists spoke about the historical marginalisation of the province in which she worked and the impact this had on infrastructure and service delivery. Challenges to the latter were echoed by others and contributed to environmental barriers to patients accessing care, particularly in bad weather or when there was violence in communities. Participants spoke of encountering trauma and abuse and the high incident of injuries caused by interpersonal violence.

Unemployment affected all participants' practice. Injury-on-duty for manual labourers was common, and these patients were often employed casually. Patients would often not attend their hospital appointments to avoid losing their jobs, even when their injury had been sustained on the job. High levels of unemployment also came with a demand to assess eligibility for disability grants. Study participants perceived a need to engage in an advocacy role to prevent injuries and address poor labour conditions and the underlying social determinants of health and social and occupational injustice.

Poverty was also evident in the occupations of patients and communities: occupations were sparse or “unfulfilled” and resource-scarce environments provided restricted opportunities for engagement. However, despite this perceived deprivation, participants noticed the deep meaning and beauty that simple occupations held.

#### 3.1.2. Strained System

“This (the strained system) was the centre of my experience…I often felt I was spending more time fighting the system than actually working with patients.” Tired & Trying

This subtheme resonated with all participants, although *Bad Grad* clarified that her experience of the strained system at her large, urban hospital was “not as drastic” as resources and expertise were more readily available there.

Participants commonly reported a poor work ethic and a staff culture of complacency at work despite some staff being friendly and helpful. Despite some facilities being clean and neat, space in facilities to treat patients was a common problem. Patients had mixed experiences of healthcare; some patients reportedly mistrusted Western medicine and the hospital represented a place where you go to die. Participants reported needing to respond to the preconceptions that patients or staff had about them, demanding that they prove themselves within their role. Navigating relationships with colleagues and teamwork was challenging at times.

Working in the strained system placed numerous professional demands on participants including being assertive, resolving conflict skilfully, working independently, having patience and perseverance, and being adaptable. Being politically aware, having a knowledge of legislation and policies, and being able to align services with the district and primary health care plan, as well as having to report on services to management, were further perceived demands.

#### 3.1.3. Contraptions and Contractures

This subtheme captures the poor medical and surgical management of hand injuries. One participant shared her frustration when a doctor had attempted to fabricate a Kleinert splint using padded aluminium splinting strips on the patient's wrist, syringe needles attached to the patient's fingernails, and elastic bands connecting each fingernail to the wrist splint:

“I had a patient walk into my office with some DIY dynamic splint made by a doctor that's not even…an orthopaedic surgeon, who did a tendon repair bedside…He (the patient) walked in with this contraption, like (a) very brief doctor's note and contractures in… 3 fingers. The wound went septic... It's very, very frustrating because I feel like the doctors... are also like guessing what to do with these patients and not managing them properly.” The Growing Therapist

Medical management of hand injuries was largely inadequate. Insufficient doctors and problematic referral systems were common complaints. Orthopaedic services were limited, late presentation of injuries was common, and secondary complications were prolific.

#### 3.1.4. Insurmountable Need

Participants were also submerged in an experience of insurmountable need. Two participants articulated aspects of this.

“Nobody in the hospital… can fix all these peoples' problems” The Bad Grad.

“The job makes me feel hopeless at times when I cannot help with the multitude of needs with my OT knowledge.” Eager and Willing

Managing a diverse caseload contributed to the sense of need. Feelings of having “no idea” and being eager and anxious were common, as well as the sense of helplessness that came with being unable to help. Feeling challenged, angry, and frustrated was common.

The environment demanded empathy, participants commonly reported feeling tired and emotionally exhausted, and some felt at risk of burnout. One participant expressed the following on completion of her community service year:

“Burnout is real!” Solo Worker Bee

Amidst these realities, participants agreed that they needed to be able to prioritize needs, understand their own limitations, and have emotional intelligence. Good time management and conscientious hard work were demanded. They also had to be resilient and able to cope with stress, frustrations, and challenges. The realities also contributed to a need to be affirmed, heard, and understood. Participants shared how they tried to stay positive, found satisfaction in making small differences, and learnt to celebrate progress.

#### 3.1.5. Engaging through Diversity

Diversity was another salient feature of the context in which participants were submerged. Language and cultural differences were most often mentioned, and both the challenges and personal growth associated with this experience of diversity were shared. One participant noted that in her urban practice setting, language and cultural differences were not as pronounced, rendering language barriers easier to overcome. This contrasts with the rural participant who compared her experience to her urban undergraduate experience.

“Thinking back on patients I saw as a student, it amazes me like, we actually had a conversation whereas I haven't really had a full conversation with a patient this whole year. Some people can speak a little bit of broken English, but that's about it. So that really affects... even just the interpersonal relationship that you can build with a patient it's very limited which makes (one) feel quite isolated from one's patient, so it's difficult to connect.”

As reflected in the quote, communication challenges were very common, placing demands on therapists to converse in the patient's language. Cultural barriers existed and necessitated an understanding and cultural humility from participants. Mention was made of the need to respond well to patients' traditional health beliefs and practices. The need for cultural humility resonated strongly with one of the participants,

“I had to be reflective and make a concerted effort to be aware of my cultural blinders such that I can recognise… (our) shared humanity.” Dedicatedly Winging-it

Participants described many aspects in which their experience was different from “what I know”. One participant explained how her prior exposure to diversity through family life had prepared her for difference, but for others, fitting in was a challenge. Despite this difference, participants marvelled at the sense of community that they witnessed and saw the beauty in the natural environment as well as the beauty of human participation in everyday occupation. Participants expressed gratitude and a sense of having grown and gained invaluable learning and life experience through their CS experience.

### 3.2. Theme 2: Starting Somewhere

This theme captures characteristics of the process or journey that participants experienced in delivering hand-injury care. The name of the theme is taken from *Bad Grad* who said that “everyone has to start somewhere”. Five subthemes describe this journey.

#### 3.2.1. No Idea to Some Idea

The journey of delivering hand-injury care was often started with a sense of having no idea:

“So now I'm kind of stuck. I have no idea where to go with this hand. She is coming back sometime this week and then I need to do something. And I have no idea what to do.” Tired and Trying

Exclamations of “what is this?” and desperate questions of “what do I do?” were common. Participants typically felt overwhelmed in the beginning, finding the task of hand therapy daunting. Some reflected on feeling as if they had initially done more harm than good. A lack of confidence, a sense of inexperience, and questioning their competence were common.

The sense of “no idea” was, however, not terminal: participants demonstrated that they were willing and trying to find their feet. A gradual orientation towards “starting somewhere” was achieved. This came with a surprising sense of “doing better than one thinks.” Gradually, participants acclimatised to thinking on their feet and experienced success and satisfaction in being able to “do something.”

#### 3.2.2. Finding Directions

This subtheme captured the challenge of clinically reasoning around hand injuries as an inexperienced therapist submerged in the contextual realities outlined in theme 1. One participant explained.

“I obviously didn't know much about hands at all, but I am an avid Googler…I am not shy of taking out my phone and looking up what or how or why. But there's also this thing about the lack of diagnosis....” Anx-cited Outsider

In addition to the challenge of unfamiliar conditions, this quote captures the compounded challenge of “finding directions” when patients have no diagnosis. Participants were required to clinically reason around chronic conditions as well as acute and traumatic injuries. They were frequently required to treat patients with fractures, poststroke upper limb impairment, and burns to the hand.

These demands were accompanied by a desire for learning opportunities, a need to learn by doing, as well as a desire to share their knowledge and experiences with others in similar contexts. They reported a need for knowledge and skills, along with support for developing basic hand therapy skills. They frequently sought guidance to aid their clinical reasoning, and feedback to consolidate and learn from their actions.

#### 3.2.3. I Know There Is a Protocol but…

This subtheme communicates the complicated presentations and the advanced demands that this places on the therapist's reasoning. These “curve-ball” presentations are largely complications associated with contextual features.

“And then when a hand injury will come in that doesn't suit a certain box or like a certain picture.” Tired and Trying

With protocols usually outlining typical presentations and timeframes, these were less useful when patients presented long after surgery or were never able to access surgery. Numerous challenges to follow-up appointments with patients and clinic outreach demanded mobile or travelling hand therapy services. Patient adherence was often problematic, demanding appropriate intervention guidelines, education, and home programmes that were responsive to these realities.

#### 3.2.4. Sparse Equipping

Participants' journey of delivering hand-injury care was further characterised by sparse equipping on many fronts. Dedicatedly Winging-it explained some of these.

“I am the only OT (at my) Community Health Clinic there's no permanent staff... since 2017 in rehab... It's quite hectic... it's quite a busy clinic... I remember the first few weeks, was the most overwhelming thing I've ever experienced... especially with hands...(I) went from having absolutely no clinical experience (at university) ... And now … this three-year-old child that's just (burnt) his fingers, and now I must do stuff…It felt so unethical and I'd … run around trying to call my supervisor who's … in (a district) hospital and she's obviously also trying to be a therapist there, and finding out what I must do.” Dedicatedly Winging-it

As evidenced in this quote, undergraduate preparation was considered by some to be inadequate with a lack of practical or fieldwork experience in hand rehabilitation being frequently highlighted. Supervision by more experienced clinicians was often absent or unsatisfactory. While therapists with supervision were grateful for any supervision, it was identified that this could be both an opportunity (for learning and support) but also a barrier to feeling free to explore therapy without a sense of being watched. Practice conditions were largely accompanied by a need for support and a need for mentoring and supervision. Reflection as a way of “learning from yourself” was considered vital, demanding the necessary reflective practice skills to achieve this.

A number of participants who were the only occupational therapist in their setting found this to be challenging. Unsurprisingly, many expressed a need for colleagues and for the creation of occupational therapy jobs so that they would not be alone.

Existing courses were often not well suited to need, highlighting a gap for appropriate continuous professional development (CPD) opportunities. Circumstance also called for therapists to know how to search for evidence. Limited physical resources and inadequate hand therapy equipment were common. Although this provided an opportunity to nurture their resourcefulness and creativity, the need for resources was clear: appropriate splinting equipment, appropriate consumables (e.g., splinting material), versatile resources that could be used for a diverse generalist caseload, and appropriate assessment and treatment resources. Participants articulated a need for an organized treatment station that was portable and ergonomic.

#### 3.2.5. Rooted in Occupation

This subtheme captures another dimension of participants' journey: the consolidation of their appreciation of occupational therapy for patients with hand injuries. Some participants started the journey perceiving a disjuncture between hands and occupational therapy.

“So, I guess as a student I kind of felt that hands was kind of more physio-ish. I didn't really see it as being that OT.” Illusionless Optimist

For this therapist and a few others, there was an initial dislike or disinterest in hands. However, their journey of hand-injury care in CS was integral to appreciating the role of occupational therapy in hand rehabilitation and the link between the hand and occupation.

Participants endeavoured to understand what occupations people use their hands for. In rural contexts, this meant understanding and being able to analyse unfamiliar occupations, like cindering a floor (a process of sealing and polishing the floor of a hut using a mixture of cow dung and mud, typically performed in four-point kneeling). This learning was accompanied by a need to “figure out” activity and occupation and to deliver therapy that was occupation-based. This demanded resources for occupation-based hand therapy and a desire for activity ideas so that they did not feel like they were using the same activity with every patient.

### 3.3. Theme 3: “Dynamics of Surthrival”

The third and final theme sought to capture various dynamics that either supported or enabled growth and learning and a strong response to the demand for hand-injury care or dynamics that hindered this. The theme resonated strongly with participants, one of which explained it as “the transition between thriving and just surviving.”

#### 3.3.1. Propelled by Demand

The first subtheme interpreted the *diverse caseload* that participants had as an antecedent for growth: the demand for hand-injury care forced them to learn. Their experience of engaging in the task of hand-injury care was, however, a dynamic experience, accompanied at times by more or less of an experience of thriving or surviving. Illusionless Optimist expressed the following:

“I was just thrown in the deep end, and I was the only OT and I had to somewhat drown. Which has been negative in some ways because like there's no-one to check if you are doing things right, if you're stuck, then you're stuck. But then I think it also has been in other ways positive because it's forced me to learn and read up and like really investigate hand injuries.” Illusionless Optimist

#### 3.3.2. Agentic Momentum

An additional propelling force was the sense of agency exercised by participants to respond to the demand for hand-injury care. Participants were aware of their responsibility to deliver quality care and actively sought ways to enable this. Evidence of this included voluntarily joining the CoP and efforts to access expertise from a referral facility described in the following quote:

“So, what I actually did was I did go to (a regional hospital), I asked to join them for almost like a job shadowing, for two days just doing their hands OT. Just because in the beginning I was very overwhelmed. With how many hands patients and feeling very inexperienced.” Solo Worker Bee

Further evidence of agency was seen in participants taking initiative, immersing themselves in their context, researching, and reflecting. They established support networks, sought support and expertise, made attempts to cross language barriers, and embraced learning-by-doing. They negotiated the need for reasoning around hand injuries through problem-solving, thinking on their feet, and going back to basics. When unsure of how to proceed with a patient, they contained their uncertainty by focusing on clients' occupational participation and function. Exercising agency was further evidenced in participants identifying their own support and development needs.

#### 3.3.3. Support for Learning

This subtheme communicates the supports that enabled growth and a shift towards thriving. Participants considered it important to appreciate that the inverse was often true when supports were not in place.

As evidenced by this quote, case discussion and support for clinical reasoning were experienced as helpful.

“The most valuable aspects of her session was talking through cases and getting advice on how to approach them. This helped to grow my clinical reasoning as well as provide mentorship that was desperately needed.” Anonymous

In addition, undergraduate preparation, reflecting and discussing with colleagues, appropriate courses, social media, and relationships with doctors were identified as additional supports for learning in hand-injury care.

#### 3.3.4. Resources

The absence or presence of physical resources for hand rehabilitation and the presence or absence of upreferral resources were significant in the participants' experience. Solo Worker Bee shared,

“I referred (the patient to the regional hospital) just because I honestly didn't know what to do with them.”

By contrast, the Find-a-way OT shared,

“(I) was as not able to refer as I was the up referral… unless I sent them to a provincial hospital, but because of the resources (travel and others) it was never possible for a patient to go there, and the hospital was also not always able to see them due to their own high patient numbers. So I never had any patient actually be upreferred.”

Similarly, participants shared contrasting experiences around the availability of treatment modalities and physical resources for hand rehabilitation.

#### 3.3.5. Emotional Supports

The need for emotional supports is captured in this subtheme. Participants shared that becoming desensitized or dissociating were common protective emotional responses with some participants using debriefing and the setting of boundaries to cope emotionally. Emotional support was largely accessed through family and friends, colleagues, and other CSOTs. The affirmation received through patient feedback also acted as an emotional resource. Some participants indicated that staying home or being able to visit home was a support. One participant felt that if she had been able to stay home and access her usual supports, this would have been an enabler. Exposure to abuse and violent crime made emotional support essential and one participant shared.

“(I) needed a psychologist to make it through the year.” Tired and Trying

#### 3.3.6. Catalysts

Immersed in the data over an extended period made apparent to the researcher that there were certain factors that “hit-the-spot” in terms of participants' support and development needs. These factors acted as catalysts for moving in the direction of “thriving.” One catalyst, the presence of immediate or real-time support, is evidenced in the following quote:

“Access to mentorship programmes and real-time support/CoP is a wonderful benefit!” Eager and Willing

Although peer support was used, having access to expertise was considered invaluable. The proximity of support mattered: in situ support was desired. Support that was specific or responsive to emerging needs was also valued over generic input or teaching. The CoP as a mode of learning and support also proved to be a catalyst for thriving due to its versatility in meeting many of the needs articulated by participants.

### 3.4. Proposed Interventions to Address Support and Development Needs

The proposed interventions identified through participants' experiences are listed in Table 4 and discussed in the next section. The subthemes from which each intervention was extrapolated are indicated in brackets in Table 3.

## 4. Discussion

This study is aimed at describing the experiences of novice occupational therapists delivering hand rehabilitation in order to identify their support and development needs. An extensive list of proposed interventions was developed, key aspects of which are discussed in this section.

### 4.1. System and Service-Related Interventions

The pervasive impact of poverty on the practice and experiences of participants necessitates action that extends beyond the health sector alone. Global [44] and national [45] development goals highlight the need to address the social determinants of health (e.g., decent work and fair employment; income and wealth; and healthcare [46]) and the intersectoral action that is required to do so [47]. Occupational therapy's expertise in understanding the relationship between people (individual/collective), occupation, and the environment, as well as the social and occupational injustices that may be entrenched and enacted through this transaction [48], makes occupational therapists invaluable to systemic development. In South Africa, the ongoing and urgent need to position occupational therapists in all government sectors to influence and implement policy requires the occupational therapy body to renew the reputation of the profession through evidence-supported advocacy. This need is likely not limited to South Africa [49].

The strengthening of systems within the health system is also essential to supporting novice therapists in their hand rehabilitation work. Improvement of the basic management of hand injuries and conditions across the care continuum is needed with particular focus on the medical and surgical management of injuries [50]. It continues to be necessary to address the inequitable distribution of doctors in South Africa [51] as well as the shortage of orthopaedic surgeons [52]. Task shifting and support strategies hold promise [52] but need to produce quality care. The compulsory research projects conducted by medical and surgical residents in South Africa could be leveraged towards this need to ensure that the solutions developed are adequately informed by “local knowledge” (p.1080) [50]. Embedding these Master's studies within strategic, interdisciplinary, and intersectoral public health projects would be valuable in maximising their impact.

Strengthening referral systems for hand-injured patients also requires attention. Referral challenges expressed by participants were specific to their contexts and the factors that affect the functioning of health systems are known to be complex [53]. Appreciating this, the flexibility of participatory action research may be well suited to addressing specific referral system difficulties and associated problems within specific subdistricts, districts, and provinces.

Appropriate human resourcing is likewise needed to address the persistent language discordance highlighted by participants. Recruiting of occupational therapy students who reflect the language and cultural demographic of patients served by the public sector should continue to be a priority for training programs [54]. There remains, however, a need for the training and appropriate collaboration with interpreters [55] to enable adequate communication that supports quality care.

Novice occupational therapists need to be supported by access to expertise. Accessible (proximity and immediacy) support and supervision are essential, and the role of therapists with expertise or specialist training is essential to service quality and sustainability [56]. The staffing and retention of occupational therapists in the public sector continue to be problematic and require urgent attention. In the interim, innovative models for support and supervision (e.g., group mentoring and supervision, Communities of Practice) can be utilised where supervisors are compensated with continuous education units for participating in supervisor development activities and facilitating support and supervision groups. Engaging short courses that strengthen supervision capacity, increase satisfaction with supervision, and enable responsive supervision should be considered.

### 4.2. Learning Opportunities to Support Competency

Therapists require opportunities to develop the knowledge, skill, and professional behaviours across the lifelong learning continuum, which begins with strengthening undergraduate (UG) curricula. Fostering enjoyable UG experiences in hand rehabilitation [29], strengthening the occupational focus in undergraduate hand therapy teaching [57], and enabling practical hand rehabilitation opportunities [29] were highlighted in this study, as has been previously reported. This is challenging within pressured undergraduate curricula, large class sizes, and limited fieldwork placement options. Exploring the use of final-year elective fieldwork placements holds promise, providing they are carefully crafted to meet the identified learning needs. The authors plan to explore this further in a separate paper.

Regular opportunities to develop knowledge and skills were identified in this study, including the management of acute and chronic conditions and injuries, including the burnt hand, fracture management, and the poststroke upper limb. Learning opportunities to develop competencies for supporting patient adherence, accessing evidence, and assessing for disability grant eligibility are needed. Additionally, opportunities to develop agency, resilience, resourcefulness, creativity and innovation, and lifelong learning behaviours are needed. Solutions that respond to these needs should facilitate guidance and feedback that supports the development of clinical reasoning, which includes individual and group reflection and case discussions.

### 4.3. Physical Resources

Therapists cannot be expected to provide rehabilitation services without being equipped with the necessary resources. Assessment and treatment resources that can be easily carried between service sites, suitable for a diverse caseload, and that support occupation-based practice are needed. These resources need to be available, sustainably stocked, and easily procured. The participants and first author of this study developed a portable hand therapy station that is currently being trialled in two rural settings in South Africa. Feedback from this project will provide valuable data for a well-designed product and will be shared with the international therapy community to enable duplication of the practice innovation in other contexts.

In addition, there is a need for appropriate clinical guidelines that are principle-based and factor in contextual features including the absence of surgical services. Participants stated that they need appropriate home programmes, activity ideas, and continuous professional development opportunities that are suited to their level of expertise and context. Finally, mobile device applications for language learning [58] and to support communication [59, 60] between therapists and their patients should be explored to overcome language discordance.

### 4.4. Emotional Support

All themes constructed in this study spoke to participants' need for emotional support. Participants all agreed that preparation for their year of compulsory service would be valuable in developing realistic expectations and preparing oneself to access support. The pervasive need to debrief, share experiences, and receive support for reflection could be well served by online group mentorship or communities of practice.

### 4.5. Who Should Intervene?

The robust list of needs and interventions begs the question: who should intervene? Some needs are clearly the responsibility of a particular stakeholder: the Department of Health, for example, is responsible for providing equipment and resources for basic service delivery. However, many of these recommendations serve as an invitation to a diverse group of stakeholders to collaboratively, innovatively, and for mutual benefit, support novice occupational therapists in extending hand rehabilitation to populations with the greatest need for this service. Opportunity exists to look beyond the needs in South Africa and to explore international partnerships across high, middle, and low-income contexts to address common challenges to hand therapy service provision and support the cadre of therapists that deliver these services globally.

### 4.6. Strengths and Limitations

This study is the first to identify support strategies for novice generalist occupational therapists required to provide hand rehabilitation services in underserved contexts. Robust strategies were used to strengthen the trustworthiness of results, although a somewhat limited description of individual participants and their contexts may limit readers' ability to assess the transferability of findings to other practice contexts. Diverse data collection techniques proved challenging to manage but provided an opportunity for data triangulation and strengthened the benefits that participants were able to derive from the research.

## 5. Conclusion

This study elicited not only richly descriptive experiences of community service occupational therapists doing hand therapy in underserved South African contexts but also proposed strategies for the support and development of novice, generalist occupational therapists delivering hand rehabilitation in these settings. Three themes communicate participants' experience of delivering hand rehabilitation as novice generalist occupational therapists: (1) “submerged: I had to drown a little” illustrates key contextual features that characterised their experience including pervasive poverty and a struggling health system. (2) “Starting somewhere” conveys participants' journey of treating patients with hand injuries. They transitioned from an initial sense of having “no idea” to developing “some idea” while being challenged by unfamiliar presentations, no diagnoses, or traditional approaches to therapy proving inappropriate for their settings. Finally, (3) “dynamics of ‘surthrival'” captured aspects that enabled thriving or contributed to merely surviving hand rehabilitation. Key areas for intervention were identified from participants' experiences; systems and health service interventions; opportunities to develop the necessary knowledge, skill, and professional behaviours; appropriate resources; and emotional supports. These strategies may be considered for supporting generalist or novice therapists delivering hand rehabilitation in other underresourced contexts.

## Figures and Tables

**Table 1:** Profile of the occupational therapist participants.

	Pseudonym	Location (level of healthcare facility, urban/rural setting, province)	No. hand-injured patients seen monthly
1	Illusionless Optimist	District hospital, rural, Eastern Cape	25
2	Dedicatedly Winging-it	Primary clinic, rural, KwaZulu Natal	30
3	Eager & Willing	Primary clinic, periurban, Gauteng	8
4	The Bad Grad	Regional hospital, urban, Gauteng	100^∗^
5	Tired & Trying	District hospital, rural, Eastern Cape	40
6	Anxi-cited Outsider	Regional hospital, rural, Mpumalanga	32
7	Find-a-way OT	Regional, rural, Limpopo	32
8	Solo Worker Bee	Primary clinic, urban, Gauteng	40
9	The Growing Therapist	District hopistal, rural, Mpumalanga	20

^∗^On orthopaedic rotation.

**Table 2:** Description of how data collection techniques were used.

Data collection tools and techniques	Procedure
Demographic online questionnaire	Participants provided basic demographic and practice data using an online survey (MS Forms)
Photoelicitation: “sharing experiences”	Participants shared photographs that captured aspects of their experience. Descriptions of the photographs and resulting group discussions were audio-recorded and transcribed.
Facilitated reflection using sentence stems [36]: “me in my setting”	Participants were given a page of sentence stems that prompted reflection on their community service contexts and their experience thereof. Completed sentences were shared with the group and uploaded in Word files to NVivo for analysis.
Authoring a congress abstract	Participants, along with the first author, wrote and submitted an abstract (title: *Hand therapy on the move – taking therapy to harder-to-reach communities*) that presented a practice innovation (portable hand therapy station) that the group planned to develop together. The abstract was uploaded to NVivo for analysis.
Facilitated reflection using the person-environment-occupation model [37]	Participants reflected on their experience of delivering hand rehabilitation using the person-environment-occupation model, reflecting on aspects of the occupation (delivering hand rehabilitation), the environment (complex healthcare context), and the person (their personal experiences, knowledge, skills, etc.) that influenced their experience across their community service year. Participants illustrated this transaction on paper and explained their experience to the group. Photos of the illustrations and transcribed audio recordings of the explanations were uploaded for analysis.
Real-time troubleshooting: case discussions on WhatsApp	The need to include a platform for “real-time” support emerged early in the study, and additional ethical permission was obtained to include WhatsApp as a data collection tool. Informal case discussions occurred on this platform as participants sought guidance and feedback.
Feedback on professional development activities	Experts were invited to the CoP based on emerging needs in the group. This also enabled the first author to focus on her role as a researcher. These sessions were evaluated in an online survey (MS Forms), and the data from the survey was uploaded to NVivo for analysis. (Professional development activities included “Clinical reasoning 101”; formal case discussions; basic biomechanical and treatment principles; management of the neurologically impaired hand; sensory reeducation and considerations for disability grant eligibility; and occupation-based hand therapy)
Online (MS Forms) evaluation experience	An online survey questionnaire was developed to evaluate participants' experience of the CoP. Data were downloaded to Excel and Word and uploaded to NVivo for analysis.
Workshop to prepare congress presentation	Participants' abstract was accepted. A hybrid workshop was thus arranged to develop the practice innovation. Participants, supported by the first author, systematically reflected on the occupational roles that their hand-injured clients engaged in; the contextual realities of their hand rehabilitation practice; and photographs of their community service year. Participants also reviewed local hand rehabilitation evidence. This data, used to inform the design of their practice innovation, was collaboratively developed on a Google Doc. A copy of the document was uploaded to NVivo for analysis.
Member checking	Analysis findings were presented to participants at intervals throughout the study. Feedback from these sessions was included as data for further/final analysis.

**Table 3:** Composite vignette of participants' experience of delivering hand-injury care.

“Welcome to my corner of the hand therapy world. It's beautiful – is not it? The rolling green hills dotted with rondavels (round mud huts) and grazing animals. Shacks and spaza shops (informal convenience shops) line the dirt road as I approach the hospital. The effects of poverty run very deep in this community, but so does a deep sense of community life: a sharing of life in both its beauty and struggles.”
“This is a typical day: typical in that I navigated the same roads in my little Hyundai and waited patiently for the resident cows to cross our shared road. I am here completing a compulsory year of service after graduating – a strategy that my government uses towards delivering quality healthcare for all – especially rural and underserved communities.”
“We'll start the day at the hospital with the psychiatry ward round. We have the cerebral palsy group at nine and then we can head out for two clinic visits and a home visit. Let us hope that the hand patients that I've booked are able to attend their appointments. Many travel 4 hours or more to the clinic so when resources are tight, follow-up therapy is not an option.”
“My first patient, Mrs Jabavu, speaks English – this is great for me but very rare. You can just imagine how client-centred I manage to be most days gesticulating and using the 10 phrases of isi-Xhosa that I know. Collaborative goal-setting? How is that even possible without being able to communicate the basics?”
“Anyway, Mrs Jabavu tells me what happened to her hand: her husband beat her with an axe. I hold my pose. My horror. My anger for the wrongness of it all. And the fact that I will now proceed to attend to the, seemingly smallest of her problems – her lacerated hand. I scan her medical notes to understand what structures have been injured and hopefully repaired. But all I can find is, “For OT Assessment”. As usual, I'll be figuring out this diagnosis on my own.”
“I've felt overwhelmed by hands most of the year. My hand therapy knowledge and skill feel about as robust as my splinting equipment. You're on your own. It's all on you! You know nothing, or so it feels. You figure out the diagnosis and with some hope, you remember that you have a protocol for that! But this does not last long as you realise this patient had flexor tendon surgery six weeks ago and his hand remains safely wrapped in a Plaster of Paris backslab. At least that situation did not require me to make a splint with my 1.6 mm thick splinting material that expired 2 years ago in a frying pan that has two settings: boiling point and off. Anyway – back to Mrs Jabavu: I've learnt to acknowledge the panic and then remind myself: You know the basics. Start with the basics....”

**Table 4:** Proposed interventions to address identified support and development needs.

Identified interventions (subtheme/s from which intervention was extrapolated)
A. System/service-related interventions
(i) Systemic development (1.1) (ii) Occupational therapy human resourcing in other public sector departments (1.1) (iii) Improvement in basic management of hand injuries/conditions (1.3) (iv) Strengthening human resources (medical and surgical) (1.3) (v) Strengthening referral systems (1.3; 3.4) (vi) Human resourcing of occupational therapists to reduce language discordance (1.5) (vii) Interpreters (1.5) (viii) Human resourcing (2.4) (ix) Strengthening supervision capacity (2.4) for responsive supervision (3.3) (x) Accessible (immediacy and proximity) support and supervision (3.6) (xi) Access to expertise (3.6)

B. Learning opportunities to support competency
(i) Guidance and feedback (2.1; 2.2) (ii) Support for the development of knowledge, skill, and clinical reasoning (2.1; 2.2) (iii) Accessing appropriate evidence (2.2) (iv) Management of acute conditions and injuries (2.2), chronic conditions (2.2), burnt hand (2.2), fractures (2.2), and stroke upper limb (2.2) (v) Reasoning around disability grant eligibility (2.2) (vi) Supporting adherence (2.3) (vii) Strengthening undergraduate curricula (2.4) (viii) Practical undergraduate experiences (2.4) (ix) Regular opportunities to develop knowledge and skill (2.4) (x) Strengthening resourcefulness, creativity, and innovation (2.4) (xi) Fostering enjoyable undergraduate hand therapy experiences (2.5) (xii) Strengthening occupational focus in undergraduate hand therapy teaching (2.5) (xiii) Occupation-based hand therapy opportunities (2.5) (xiv) Understanding indigenous occupations and drawing them into therapy (2.5) (xv) Supporting development of agency (3.1) (xvi) Supporting development of lifelong learning practices (3.1) (xvii) Resilience (including coping strategies, building/harnessing support networks) (3.3) (xviii) Strengthening undergraduate experiences (3.5) (xix) Individual and group reflection (3.5) (xx) Case discussion (3.5)

C. Physical resources
(i) Applications to assist with language learning and to support communication (1.5) (ii) Appropriate guidelines for managing conditions (2.2; 2.3) (iii) Appropriate home programmes (2.3) (iv) Mobile assessment and treatment resources (2.3) (v) Appropriate hand therapy resources (2.4) (vi) Resources suitable for a diverse caseload (3.2) including resources for occupation-based therapy (2.5) (vii) Activity ideas (2.5) (viii) Appropriate resources (3.4) (ix) Appropriate courses (3.5)

D. Emotional support
(i) Preparation for CS to know what to expect (all themes) (ii) Processing experiences: debriefing/sharing (all themes) (iii) Supported reflection (all themes)

## Data Availability

Data is available on reasonable request from the first author.

## Supplementary Materials

Supplementary 1 Positionality statement of first author.

Supplementary 2 Record of member checking conducted with all participants.

Supplementary 3 Report of analysis process submitted for auditing.

## References

[1] Dias J. J., Garcia-Elias M. (2006). Hand injury costs. *Injury*.

[2] Colen D. L., Fox J. P., Chang B., Lin I. C. (2018). Burden of hand maladies in US emergency departments. *The Hand*.

[3] Larsen C. F., Mulder S., Mette A., Johansen T., Stam C. (2014). The epidemiology of hand injuries in the Netherlands and Denmark. *Injury Epidemiology*.

[4] Siotos C., Ibrahim Z., Bai J. (2018). Hand injuries in low- and middle-income countries: systematic review of existing literature and call for greater attention. *Public Health*.

[5] Corlew D. S., McQueen K. A. K. (2017). International Disease Burden of Hand Burns: Perspective from the Global Health Arena. *Hand Clinics*.

[6] Beveridge M., Howard A. (2004). The burden of orthopaedic disease in developing countries. *Journal of Bone and Joint Surgery*.

[7] Robinson L. S., Sarkies M., Brown T., Hons B., Ot C., Brien L. O. (2016). Direct, indirect and intangible costs of acute hand and wrist injuries: A systematic review. *Injury*.

[8] McGuire D. (2018). Hand surgery in South Africa. *The Journal of Hand Surgery, European Volume*.

[9] Norman R., Matzopoulos R., Groenewald P., Bradshawa D. (2007). The high burden of injuries in South Africa. *Bulletin of the World Health Organization*.

[10] Moultrie T. A., Dorrington R. E., Laubscher R. (2021). Unnatural deaths, alcohol bans and curfews: evidence from a quasi-natural experiment during COVID-19. *South African Medical Journal*.

[11] Matzoupolos R., Walls H., Cook S., London L. (2020). South Africa’s COVID-19 alcohol sales ban: the potential for better policy-making. *International Journal of Health Policy and Management*.

[12] Jones E., van Stormbroek K. COVID-19 lockdown in South Africa: the impact of alcohol restrictions on hand injuries.

[13] Schultz G., Mostert K., Rothmann I. (2012). Repetitive strain injury among South African employees: the relationship with burnout and work engagement. *International Journal of Industrial Ergonomics*.

[14] Jeebhay M., Jacobs B. Occupational health services in South Africa. Vol. 29, South African health review. https://www.hst.org.za/publications/SouthAfricanHealthReviews/sahr1999.pdf.

[15] Makobore P., Galukande M., Kalanzi E., Kijjambu S. C. (2015). The burden of hand injuries at a tertiary hospital in sub-Saharan Africa. *Emergency Medicine International*.

[16] Stewart A., Biddulph G., Firth G. (2017). The aetiology of acute traumatic occupational hand injuries seen at a South African state hospital. *South African Orthopaedic Journal*.

[17] Ahmed E. (2010). The management outcome of acute Hand Injury in Tikur Anbessa University Hospital, Addis Ababa, Ethiopia. *East and Central African Journal of Surgery*.

[18] South African National Department of Health (2017). *National Health Insurance: Towards universal health coverage*.

[19] van Stormbroek K. (2015). *Hand-Care for All: Towards Strategic Conversations*.

[20] Mennen U., van Velze C., Mennen U., Velze C. (2008). *The Hand Book*.

[21] van Biljon H. (2016). *Transforming the Vocational Rehabilitation Services of Occupational Therapists in Gauteng Public Healthcare through Action Learning Action Research*.

[22] World Federation of Occupational Therapists Occupational Therapy Human Resources Project. https://wfot.org/resources/occupational-therapy-human-resources-project-2020-alphabetical.

[23] World Bank *Poverty and Equity Brief: Sub-Saharan Africa-South Africa*. http://www.worldbank.org/poverty.

[24] Africa S. S. (2023). *Quarterly Labour Force Survey*.

[25] Rabiul I. S. (2017). Acute occupational hand injuries with their social and economic aspects: a hospital based cross sectional study. *Orthoplastic Surgery & Orthopedic Care International Journal*.

[26] American Society of Surgery of the Hand (ASSH) Hand Therapy. http://assh.org/handcare/What-is-a-Hand-Therapist.

[27] Nandgaonkar P. (2014). The experience of being a hand therapist in India. *Journal of Academia and Industrial Research (JAIR)*.

[28] van Stormbroek K., Buchanan H. (2019). Novice occupational therapists: navigating complex practice contexts in South Africa. *Australian Occupational Therapy Journal*.

[29] van Stormbroek K., Buchanan H. (2017). Novice therapists in a developing context: extending the reach of hand rehabilitation. *Hand Therapy*.

[30] Rural Health Advocacy Project Rural Health Fact Sheet 2015. http://www.rhap.org.za/wp-content/uploads/2015/09/RHAP-Rural-Health-Fact-Sheet-2015-web.pdf.

[31] Reid S. J. (2001). Compulsory community service for doctors in South Africa - an evaluation of the first year. *South African Medical Journal*.

[32] Baxter P., Jack S. (2008). Qualitative case study methodology: study design and implementation for novice researchers. *The Qualitative Report*.

[33] Braun V., Clarke V. (2021). One size fits all? What counts as quality practice in (reflexive) thematic analysis?. *Qualitative Research in Psychology*.

[34] Kwa Zulu-Natal Province Department of Health Referral System-Levels of Care. http://www.kznhealth.gov.za/Referral-system.htm.

[35] Glaw X., Inder K., Kable A., Hazelton M. (2017). Visual methodologies in qualitative research. *International Journal of Qualitative Methods*.

[36] Reed J., Koliba C. (1995). *Facilitating Reflection: A Manual for Leaders and Educators*.

[37] Law M., Cooper B., Strong S., Stewart D., Rigby P., Letts L. (1996). The person-environment-occupation model: a transactive approach to occupational performance. *Canadian Journal of Occupational Therapy*.

[38] Braun V., Clarke V. (2006). Using thematic analysis in psychology. *Qualitative Research in Psychology*.

[39] Terry G., Hayfield N., Clarke V., Braun V., Braun V., Willig C. (2017). Thematic analysis. *The SAGE Handbook of Qualitative Research in Psychology*.

[40] Spalding N. J., Phillips T. (2007). Exploring the use of vignettes: from validity to trustworthiness. *Qualitative Health Research*.

[41] Krefting L. (1991). Rigor in qualitative research: the assessment of trustworthiness. *American Journal of Occupational Therapy : Official Publication of the American Occupational Therapy Association*.

[42] Darwin Holmes A. G. (2020). Researcher positionality - a consideration of its influence and place in qualitative research - a new researcher guide. *Shanlax International Journal of Education*.

[43] Olmos-Vega F. M., Stalmeijer R. E., Varpio L., Kahlke R. (2023). A practical guide to reflexivity in qualitative research: AMEE guide no. 149. *Medical Teacher*.

[44] World Health Organization Sustainable Development Goals. https://sustainabledevelopment.un.org/?menu=1300.

[45] National Planning Commission, Department of the Presidency R of SA National Development Plan -2030. https://www.gov.za/sites/default/files/gcis_document/201409/ndp-2030-our-future-make-it-workr.pdf.

[46] Marmot M., Bell R. (2018). The sustainable development goals and health equity. *Epidemiology*.

[47] McKenzie A., Schneider H., Schaay N., Vera Scott D. S. Primary health care systems: case study from South Africa. https://www.who.int/alliance-hpsr/projects/alliancehpsr_southafricaprimasys.pdf?ua=1.

[48] World Federation of Occupational Therapists (2016). *Minimum Standards for the Education of Occupational Therapists*.

[49] Carey A., Lynn Cockburn S. L. (2019). Investigating the provision of occupational therapy services: a case study. *Canadian Journal of Occupational Therapy*.

[50] Dias J. J., Chung K. C., Garcia-Elias M., Sabapathy S. R., Tang J. B. (2006). Recommendations for the improvement of hand injury care across the world. *Injury*.

[51] Mofolo N., Heunis C., Kigozi G. N. (2021). Corrigendum: towards national health insurance: alignment of strategic human resources in South Africa. *African Journal of Primary Health Care & Family Medicine*.

[52] Ntambue J. K., Groenewald J., Arnolds D. (2019). WhatsApp mobile health platform to support fracture management by non-specialists in South Africa. *Journal of the American College of Surgeons*.

[53] Malakoane B., Heunis J. C., Chikobvu P., Kigozi N. G., Kruger W. H. (2020). Public health system challenges in the Free State, South Africa: a situation appraisal to inform health system strengthening. *BMC Health Services Research*.

[54] Ned L., Tiwari R., Buchanan H., Van Niekerk L., Sherry K., Chikte U. (2020). Changing demographic trends among South African occupational therapists: 2002 to 2018. *Human Resources for Health*.

[55] Benjamin E., Swartz L., Chiliza B., Hering L. (2016). Language barriers in health: lessons from the experiences of trained interpreters working in public sector hospitals in the Western Cape. *South African Health Review*.

[56] World Federation of Occupational (2014). *Position statement: specialisation and advanced occupational therapy competencies*.

[57] van Stormbroek K., Buchanan H. (2018). Hand health for all: do undergraduate occupational therapy hand curricula respond to the call?. *South African Journal of Occupational Therapy*.

[58] Heil C. R., Wu J. S., Lee J. J., Schmidt T. (2016). A review of mobile language learning applications: trends, challenges, and opportunities. *EuroCALL Review*.

[59] Müller F., Chandra S., Furaijat G. (2020). A digital communication assistance tool (DCAT) to obtain medical history from foreign-language patients: development and pilot testing in a primary health care center for refugees. *International Journal of Environmental Research and Public Health*.

[60] Ji X., Chow E., Abdelhamid K. (2021). Utility of mobile technology in medical interpretation: a literature review of current practices. *Patient Education and Counseling*.

