# Supplementary material for: “Surthriving” Hand Rehabilitation: Proposing Interventions to Support Novice Occupational Therapists Working in Underserved Contexts
Source: Occup Ther Int. 2023 Dec 14;2023:5562025. doi: 10.1155/2023/5562025 (PMC10735728; doi:10.1155/2023/5562025)
Supplement: Supplementary 1 — Positionality statement of first author. [file 5562025.f1.docx]

**Supplementary file I**

The first author’s personal experience of delivering hand rehabilitation as a novice practitioner in 2006 shaped her interest in the research topic. Subsequent clinical work within a hand surgery unit and a decade of hand rehabilitation research has shaped the author’s understanding of the social determinants and consequences of hand injuries. A strong sense of justice, situated within the author’s personal worldview, has further influenced her research work towards equitable access to quality healthcare, and the needs of the practitioners that are integral to this. The South African health care context has a history of systemic oppression. Therefore, as a white, female, English-speaking researcher and educator, her justice-driven approach runs the risk of perpetuating paternalistic postures, necessitating reflexive thought and recalibration on the part of this author.

Underpinned by constructivist assumptions, the first author takes a pragmatic approach to research. This orientation has been influenced by exposure to diverse theoretical positions: early undergraduate exposure to critical perspectives in qualitative inquiry and experience of more positivist assumptions from work within clinical settings. The different research paradigms of this article’s co-authors iterate this diversity of perspectives. Occupational therapy theory has also contributed a lense through which the research question as been approached: appreciation of the person, environment, occupation transaction as a core occupational therapy assumption ^1,2^ have guided data collection and analysis.

References:

1. Law M, Cooper B, Strong S, Stewart D, Rigby P, Letts L. The Person-Environment-Occupation Model: A transactive approach to occupational performance. Can J Occup Ther. 1996;63(1):9–23.

2. King G. A framework of personal and environmental learning-based strategies to foster therapist expertise. Learn Heal Soc Care. 2009;8(3):185–99.
