# Supplementary material for: “Surthriving” Hand Rehabilitation: Proposing Interventions to Support Novice Occupational Therapists Working in Underserved Contexts
Source: Occup Ther Int. 2023 Dec 14;2023:5562025. doi: 10.1155/2023/5562025 (PMC10735728; doi:10.1155/2023/5562025)

Report on analysis process and findings

Study 2A: 23 September 2022

Thank you for considering this report for the purposes of an audit of the analysis process. Please find attached a folder containing each code with its references as well as each data source with coding stripes.

Braun and Clarke's (2006, 2020) reflexive thematic analysis (RTA) was used within the qualitative case study design. Although the phases of RTA were followed consecutively, the analysis was an iterative process. Phase 1 – 4 has been completed and is reported below. The RTA was used to answer the first part of the research question (italics) and provide the foundation for extrapolating an answer to the latter part of the question:

> *How do generalist occupational therapists (CS) describe their experience of delivering hand injury care* and what are their support and development needs?

A cross-case analysis compositional format was selected for this project. Vignettes are characteristics of case study reporting that allow the reader a vicarious experience of the findings. Given the choice of a cross-case compositional format, I have constructed a composite vignette. This vignette has been composed from the raw data: each aspect of the vignette has been drawn from at least one of the participants' account of their experience. However, all participants agreed that that the vignette, as a whole, appropriately resonated with their experience during member checking.

Composite Vignette:
Welcome to my corner of the hand therapy world. It's beautiful – isn't it? The rolling green hills dotted with rondavels and grazing animals. Shacks and spaza shops line the dirt road as I approach the hospital. The effects of poverty run very deep in this community – but so does a deep sense of community life: a sharing of life in both its beauty and its struggles.

This is a typical day: typical in that I navigated the same roads in my little Hyundai and waited patiently for the resident cows to cross our shared road. I am here completing a compulsory year of service after graduating – a strategy that my government uses towards Delivering quality *healthcare for all* – especially rural and underserved communities.

We'll start the day at the hospital with the psychiatry ward round. We have the CP group at nine and then we can head out for two clinic visits and a home visit. Let's hope that the hand patients that I've booked will be able to attend their appointments. Many travel 4 hours or more to the clinic so when resources are tight, attending follow-up therapy isn't an option.

My first patient, Mrs Jabavu, speaks English – this is great for me but very rare. You can just imagine how client-centred I manage to be most days gesticulating and using the 10 phrases of isi-Xhosa that I know. Collaborative goal-setting? How is that even possible without being able to communicate the basics?

Anyway: Mrs Jabavu tells me what happened to her hand: her husband beat her with an axe. I hold my pose. My horror. My anger for the wrongness of it all. And the fact that I will now proceed to attend to the, seemingly, smallest of her problems – her lacerated hand. I scan her medical notes to understand what structures have been injured and hopefully repaired. But all I can find is: "For OT Assessment". And as usual, I'll be figuring out this diagnosis on my own.

I've felt overwhelmed by hand patients most of the year. My hand therapy knowledge and skills feels about as robust as my splinting equipment. You're on your own – it's all on you! You know nothing – or so it feels. You figure out the diagnosis and with some hope, you remember that you have a protocol for that! But this doesn't last long as you realise this patient had flexor tendon surgery six weeks ago and his hand remains safely wrapped in a plaster of paris backslab. At least that situation didn't require me to make a splint with my 1.6mm thick splinting material that expired 2 years ago in a frying pan that has two settings: boiling point and off. Anyway – back to my patient. I've learnt to acknowledge the panic and then remind myself: You know the basics. Start with the basics....

Each participant gave themselves a pseudonym that captured their personas in their CS occupational therapy role. These names, along with basic demographic information for each participant, is contained in the table below:

Welcome to my corner of the hand therapy world. It's beautiful – isn't it? The rolling green hills dotted with rondavels and grazing animals. Shacks and spaza shops line the dirt road as I approach the hospital. The effects of poverty run very deep in this community – but so does a deep sense of community life: a sharing of life in both its beauty and its struggles.

This is a typical day: typical in that I navigated the same roads in my little Hyundai and waited patiently for the resident cows to cross our shared road. I am here completing a compulsory year of service after graduating – a strategy that my government uses towards Delivering quality *healthcare for all* – especially rural and underserved communities.

We'll start the day at the hospital with the psychiatry ward round. We have the CP group at nine and then we can head out for two clinic visits and a home visit. Let's hope that the hand patients that I've booked will be able to attend their appointments. Many travel 4 hours or more to the clinic so when resources are tight, attending follow-up therapy isn't an option.

My first patient, Mrs Jabavu, speaks English – this is great for me but very rare. You can just imagine how client-centred I manage to be most days gesticulating and using the 10 phrases of isi-Xhosa that I know. Collaborative goal-setting? How is that even possible without being able to communicate the basics?

Anyway: Mrs Jabavu tells me what happened to her hand: her husband beat her with an axe. I hold my pose. My horror. My anger for the wrongness of it all. And the fact that I will now proceed to attend to the, seemingly, smallest of her problems – her lacerated hand. I scan her medical notes to understand what structures have been injured and hopefully repaired. But all I can find is: "For OT Assessment". And as usual, I'll be figuring out this diagnosis on my own.

I've felt overwhelmed by hand patients most of the year. My hand therapy knowledge and skills feels about as robust as my splinting equipment. You're on your own – it's all on you! You know nothing – or so it feels. You figure out the diagnosis and with some hope, you remember that you have a protocol for that! But this doesn't last long as you realise this patient had flexor tendon surgery six weeks ago and his hand remains safely wrapped in a plaster of paris backslab. At least that situation didn't require me to make a splint with my 1.6mm thick splinting material that expired 2 years ago in a frying pan that has two settings: boiling point and off. Anyway – back to my patient. I've learnt to acknowledge the panic and then remind myself: You know the basics. Start with the basics....

1: Pseusodyms and demographic data for each participant

	Case name	Location	Province	Level of care	Hands patients/month
P1	Illusion-less optimist	Rural	Eastern Cape	District	25
P2	Dedicatedly winging-it	Rural	KwaZulu Natal	Primary	30
P3	Eager & willing**	Peri-urban	Gauteng	Primary	8
P4	Bad Grad	Urban	Gauteng	Regional	100*

P5	Tired and trying	Rural	Eastern Cape	District	40
P6	Anx-cited outsider	Rural	Mpumalanga	Regional	32
P7	Find-a-way OT	Rural	Limpopo	Regional	32
P8	Solo worker bee	Urban	Gauteng	Primary	40
P9	Growing OT	Rural	Mpumalanga	District	20

* when on orthopaedic rotation **this therapist struggled to give herself a pseudonym. This name was thus extracted from her data and she was satisfied with the choice

More detailed information for each participant is contained in appendix A.

Reflexive Thematic Analysis[1]:

Phase 1: Familiarisation with data

This phase involved listening to the audio-recordings of meetings, making notes, and reading through the transcribed audio-recordings or other text data (eg. Whatsapp group chat).

Phase 2: Systematic data coding

This phase occurred over 12 months (August 2021-September 2022) and was a *very* iterative process with multiple revisions being influenced by ongoing analysis. An inductive approach to coding was initially pursued with some deductive codes being introduced later in analysis. Both semantic (descriptive) and latent (interpretive) codes were used.

The table below tracks some of the iterations and coding decisions:

Date	Coding	Reasoning/decisions
July – October 2021	Inductive approach taken to coding	To allow data to drive analysis, to enable a broad focus and 'not miss anything'.
October-November 2021	Introduction of some deductive codes	Results of a study on the CPD needs of rural OTs in South Africa (currently unpublished) came to the attention of the researcher at this stage of data collection and analysis. Many of the 'demands' of rural practice in the latter study resonated with what the researcher was 'hearing' from participants. Thus, during an early member checking exercise, the researcher presented a list of experiences (from data), needs (from data and previously reported by CSOTs in research by van Stormbroek & Buchanaa) and demands (from rural CPD needs study). Using an electronic survey, participants rated the following:<br>• **Demands** that I've become aware of while treating hand-injured patients in Community Service (0=No; 1=Somewhat; 2=Very much)<br>• **Needs** that I perceive for delivering hand-injury care (0=No I don't experience this need, yes

		• **Experiences** around hand injury care (0=not at all; 1=somewhat; 2=very much)
21 January 2022	182 codes (9 parent codes)	At this stage the researcher included the various person, environment, and person-environment fit strategies described by King (2009) as deductive codes as the data she was observing echoed some of the strategies outlined by King.
April – July 2022	201 codes (6 parent codes)	Deductive codes from King (2009) found to be "disruptive" of overall inductive approach to coding and created somewhat artificial/cumbersome descriptions of data. These deductive codes were thus removed. Some duplicate codes were removed and some codes collapsed. Some codes were split to more accurately/descriptively capture meaning.
August - September 2022	213 (codes) 6 parent codes	Finalised code list applied throughout data. Codebook generated for audit trail purposes.

In order to "keep order", remember and navigate my growing list of codes in Nvivo, I used a number of parent codes as holders/containers. The final list of codes made use of the following six parent codes:

Broad experience
Hand-injury care experience
Demands
Needs
Supports and enablers
Strategies

Phase 3: Generating initial themes from coded and collated data

At this stage I sought to generate initial themes. It was difficult for me to do this on Nvivo so I printed out my codes in colour or wrote them on post-it notes.

Within the reflexive thematic analysis (RTA) that I was using, the following principles of the approach were evident in my analysis [1]:

- *I assumed an active role in the analysis.* In RTA, themes do not emerge but are constructed by the researcher during the familiarisation and coding phase. [rather than an archaeologist 'finding' meaning, the researcher in RTA is compared to a sculptor that constructs the image with data] [1].
- *I acknowledged my positionality, embracing my subjectivity* (as a fundamental tenet of RTA) with cognisance of enabling the reader to 'trace my subjectivity' and its influence in my report through demonstrating reflexivity. At intervals I interrogated my positionality[2], the theoretical assumptions of my work and the personal, interpersonal, methodological and contextual factors (Walsh, 2003) influencing the 'lense' through which I was viewing the data. [3]

- *I constructed themes through active pattern formation and identification:* the research question continuously guided me in terms of "what is and what is not relevant in terms of potential clusters of patterned meaning" (p.27) [1]
- *I combined and clustered codes* seeking to 'see'(generate) patterns that could be supported by the content of the codes. "Theme development involved examining codes (and associated data), and combining, clustering, collapsing together into bigger or more meaningful patterns…" (p. 28). [1]

The photographs below demonstrate stages of the 'pattern identification and generation' (between April and August 2021):

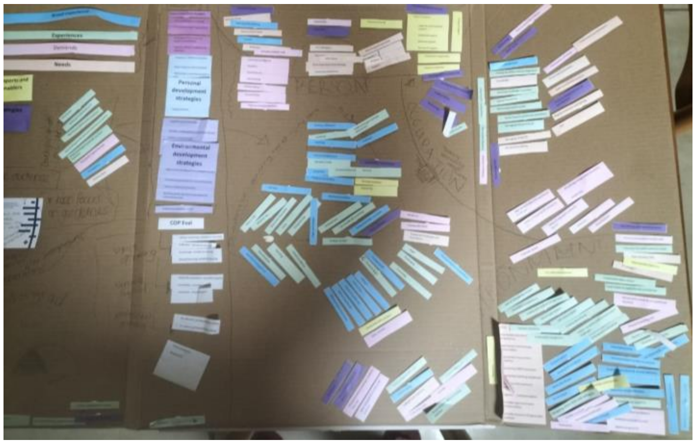

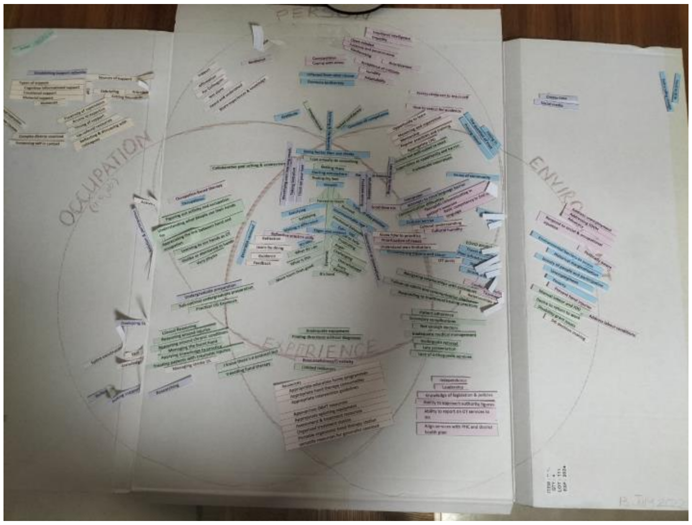

The two iterations above (April and May 2022) drew on the PEO interaction (see conceptual framework) as a guide to understanding and identifying patterns within the data. This approach, however, did not yield a meaningful understanding of the data. The iterations below simply organised / clustered similar codes (without overt use of the PEO). The clusters formed candidate "sub-themes". Similarities or links between sub-themes were interrogated until candidate themes were generated.

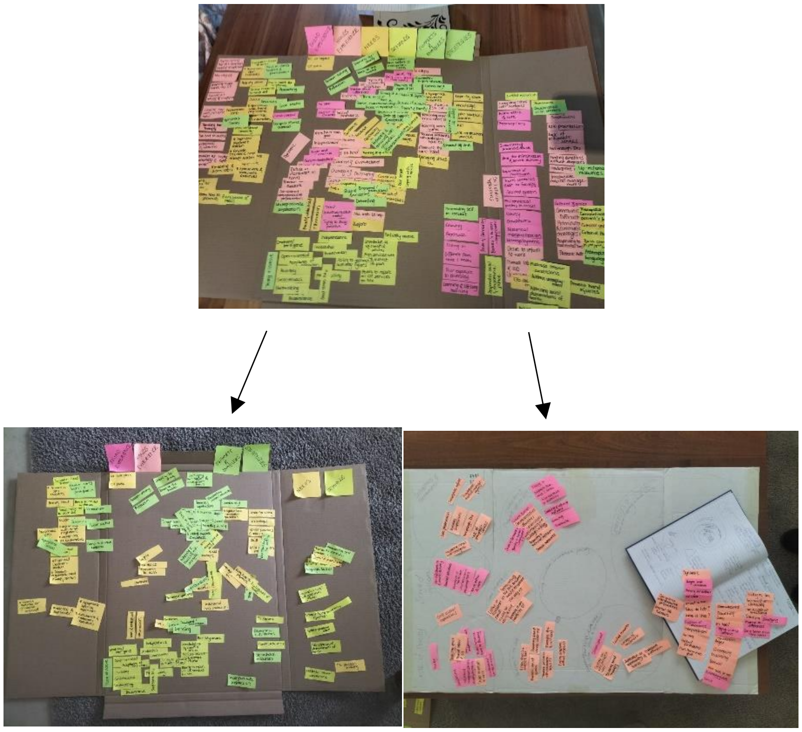

The following initial themes were presented to the project supervisors (August 2022):

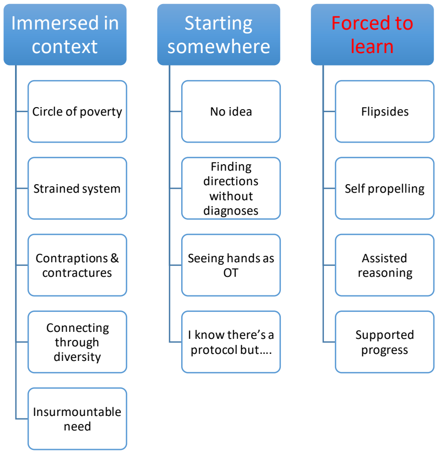

Figure 1: Initial themes (August 2022)

I felt that the third theme was particularly unrefined and 'needed work' and was thus presented in red. Based on feedback from supervisors, I returned to my posters and post-it notes to further develop, review and name my themes.

1. Terry, G., Hayfield, N., Clarke, V. and Braun, V., 2017. Thematic analysis. *The SAGE handbook of qualitative research in psychology*, *2*, pp.17-37.
2. Holmes, A.G.D., 2020. Researcher Positionality--A Consideration of Its Influence and Place in Qualitative Research--A New Researcher Guide. *Shanlax International Journal of Education*, *8*(4), pp.1-10.
3. Olmos-Vega, F.M., Stalmeijer, R.E., Varpio, L. and Kahlke, R., 2022. A practical guide to reflexivity in qualitative research: AMEE Guide No. 149. *Medical Teacher*, pp.1-11.

Phase 4: Developing and reviewing themes

<u>Member checking / member reflection</u>

At this stage of analysis, the findings (themes & sub-themes) were presented to participants in online member-checking/member-reflection meetings. Each participant indicated whether each finding

resonated with their experience by selecting *yes, no or partially* from a dropdown menu in a google document (see screenshot below). They explained or commented on their response if they chose to. After the member checking meetings and a review of all responses, the researcher contacted participants individually if additional clarity on their responses was needed. After each sub-theme is presented below, a summary of the response from members is included in a textbox along with a proposed action by the researcher (where necessary).

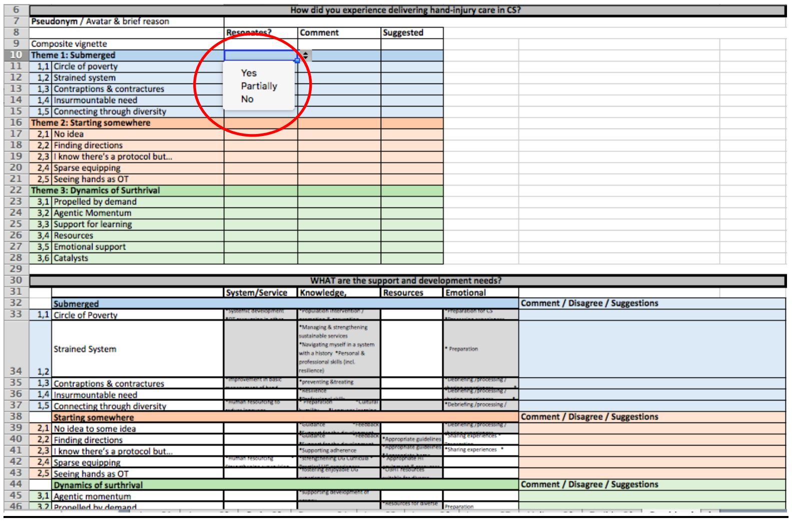

Themes and sub-themes

Three themes were generated, each with a number of sub-themes:

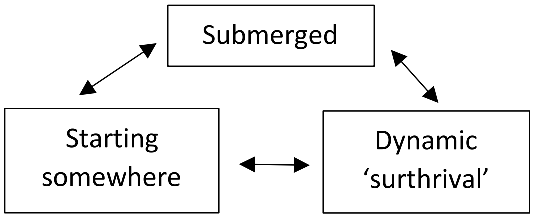

Theme 1: Submerged

A number of "code clusters" spoke to various contextual realities or features in which participants' experience was immersed or *submerged* (1.1-1.3), or were generated where codes clustered around

specific aspects of the context (1.4 and 1.5). The name of the theme (submerged) was drawn from the imagery created by *Illusionless optimist* who said, “I had to drown a little”.

The five sub-themes are illustrated below (Figure 1) as transacting circles that suspended participants’ experience.

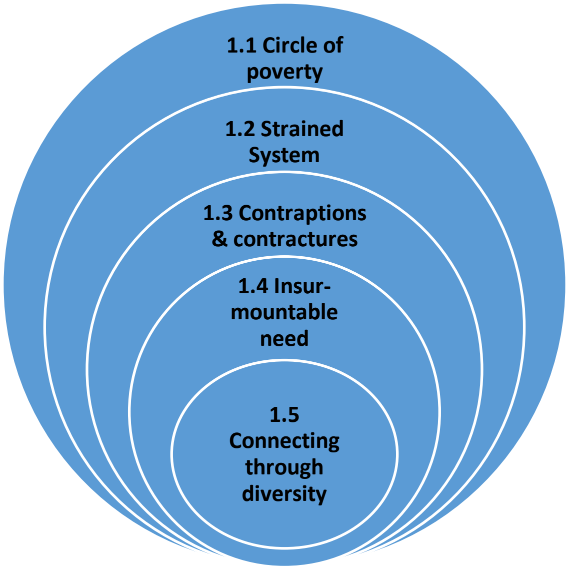

Figure 2: Five Sub-themes of Theme 1: Submerged

A summary of each of the subthemes is presented below with a table containing their codes and an extract/example quote for each code (these will not be included in the final report but have been included here to communicate the content/meaning contained in the sub-theme). The colours in the left column indicated the parent code that each code originally belonged to (Phase 2).

1.1 Circle of poverty

The first sub-theme, *circle of poverty,* captures the pervasive poverty that suspended or encircled their work:

"I didn’t understand the environment so well and because the environment was so poorly resourced... I had no idea how to go about giving hand therapy." Participant 1

Numerous references to *poverty* were made by all participants. One of the rural therapists spoke about the *historical marginalisation* of the province in which she worked and that the impact that this had on *infrastructure and service delivery*. Challenges to the latter were echoed by others and contributed to *environmental barriers to patient’s accessing care*, particularly in bad weather or when there was violence in communities. Participants spoke of *encountering trauma and abuse* and the high incident of *IPV injuries. Unemployment* affected all participants’ practice: *injury-on-duty for manual labourers* was common and these patients were often employed casually. Even if patients sustained injuries on the job, their attempt to *keep their job*, often at the expense of attending hospital appointments was common. High levels of *unemployment* also came with a demand to assess *eligibility for disability grants*. Participants perceived

a need to engage in an *advocacy* role, to *prevent injuries, address poor labour conditions*, and the *underlying social determinants of health and social/occupational injustice.*

Poverty was also evident in the *occupations* of patients and communities: occupations were sparse or "unfulfilled" and resource-scarce environments provided restricted opportunities. However, despite this perceived deprivation, participants noticed the deep meaning and beauty that simple occupations held.

	***Circle of Poverty* Codes**	**Quote sample**
	Poverty	"People's lives are characterised by…their low SES's. Their livelihood are focused on survival. Their occupations… are unfulfilled" (P4)
	Historical marginalization	"So, I work in (a) historically very marginalised (area) with poor service delivery ... a barrier that's continued to hinder service delivery... it's still very much an area of the country that is forgotten and service delivery is probably at its worst (here)... there's a barrier to people getting to the hospital, let alone just you being able to treat the patient" (P5)
	Respond to social & occupational injustice (1.75)	"His employer is only giving him an hour off to come to the hospital...the feeling that I get is that he's not a South African citizen... I almost feel as if his employer is...exploiting him a little bit... he had his injury and he had to be at work literally the next day and he's not given time off to come for therapy" (P9)
	Addressing SDOH (1.375)	"I know that there's technically legislation to protect people from not being able to work after an injury. But if they are able to work, they should go back with reasonable accommodation. But so many of the cases that our clients who work ... 1 in 200 other people that can easily be replaced by the next willing person and they often don't have that kind of legal protection, or like social mobility to make a noise. I've tried to write letters to employers and my patients will come back and say no like no improvement has been seen" (P2)
	Advocacy	
	Encountering trauma and abuse	"I have not been exposed to poverty, hardship and violence to this extent"(P9)
	Interpersonal violence injuries	"He had a stab wound because he said a gang of 15-year-old boys walking around with pangas and things and they assaulted him to get money and his phone and everything" (P3)
	Poor infrastructure & service delivery	"Like not many people have flush toilets here. We haven't had water in two months"(P1)
	Environment barriers to access	"Not many patients came through to the clinic, mostly just due to the rise (riots) as well as it being quite cold"(P8)
	Occupations	"Their occupations different to those in the city and sometimes are limited due to poor infrastructure and access to many facilities one would find in urban areas" (P9). "Their occupations are simple but have so much meaning" (P5)
	Unemployment	"Job scarcity is a big, big thing. There are so few people who actually have jobs, so many of them just sit at home and do nothing."(P6)
	Address unemployment (2)	
	Trying to stay employed	"So he's saying no, he doesn't want to wait in the line to see the specialist...Because his employer is only giving him an hour off."(P9) ("he's at least trying to not lose the job" – PI clarifies).
	Disability grant issues	"So many young men that are so capable coming and asking, saying that they're unable to work now, because they have a contracture on their one index finger" (P2)
	Manual labour & IOD	"...too many grinder injuries...I think it's a lot to do with people kind of just taking whatever work they can get...it's not like equipment that they're necessarily familiar with"(P9)
	Machinery injury	
	Address labour con ditions (1.625)	
	Prevent hand injuries (1.5)	

Member checking:		
1.1 Circle of Poverty	**No. of participants**	**Comments**
Yes, resonates	9	"Can I say super yes? This was a big factor for me" P9
Partially resonates	0	
No, does not resonate	0	

1.2 Strained system

"The system is very broken" P5 describes a strong contextual feature of their experience: working within a strained system. Although some facilities were *clean and neat*, and some *staff friendly and helpful*, participants commonly reported poor *work ethic* and a culture of complacency. *Space* to treat patients was problematic. Patients had mixed *experiences of healthcare* with some reportedly mistrusting western medicine or the hospital being thought to be the place you go to die. Participants reported needing to *respond to the preconceptions* that patients or staff had about them and sometimes demanded that they prove themselves. *Navigating relationships with colleagues* and *teamwork* could be challenging, at times demanding *conflict resolution skills*. Working in the strained system placed numerous professional demands on participants including being *assertive, working independently, having patience and perseverance, and being adaptable.*

	***Strained System* Codes**	**Quote example**
	Strained system	"The system is very broken" P 5
	Friendly & helpful staff	"The staff are friendly...willing to go out of their way to help the patients in need" P4<br>"The staff are friendly...but not hard working" P6
	Clean & neat	"The clinic building are neat and clean" P3
	Work ethic & care	"There is almost a culture of complacency at my hospital. So, we on nine staff members in the OT department, but I do most of the work and the rest of my colleagues use the, what's the word, they say that I need to see the ward patients and I need to see all of the clinic patients because I need to get the experience as the conserve." P6
	Experience of healthcare	"The community's experience of healthcare is mixed" P7<br>"The community's experience of healthcare is that nobody cares, and they come to the hospital to die." P5
	Preconceptions	"Being mixed, female and young means that often people don't want to take me seriously and can be disrespectful at times both patients and some staff members" P8
	A struggle for space	"The clinic rooms are small and it's always a struggle for space to see patients" P8
	Navigating relationships with colleagues	"I always fight with him. He doesn't like me very much. And then the other doctors like the conserves, because they know us we collaborate with them a little bit better."P9
	Conflict resolution skills (1.375)	
	Acceptance of criticism (1.5)	
	Teamwork (1.75)	"The services… can be frustrating when no one cooperates as a team"P4
	Politically aware (1.25)	

	Knowledge of legislation & policies (1.75)	
	Align services with PHC & district health plan (1.625)	
	Ability to report on OT services to management (1.375)	
	Ability to approach authority figures (2)	
	Assertiveness (1.75)	
	Leadership (1.5)	
	Work independently (1.875)	
	Patience & perseverance (2)	
	Adaptability (2)	"At the end of the year, she'll have learned how to be an OT and how to be adaptable to any situation" P6
	Open-minded (1.875)	

Member checking:		
1.2 Strained system	**No. of participants**	**Comments**
Yes, resonates	8	"This was the centre of my (CS) experience…I often felt I was spending more time fighting the system than actually working with patients"P5
Partially resonates	1	"We had more resources, more expertise…it wasn't as drastic…having a more developed hospital…there are aspects that are still a struggle…for example space and teamwork…"
No, does not resonate	0	
Action: reporting needs to capture the variance within this code to convey the Bad Grad's nuanced experience.		

1.3 Contraptions and contractures

This sub-theme captures the poor management of hand injuries as a broader context for delivering hand-injury care. One participant shared:

"He (the patient) walked in with this contraption, like (a) very brief doctor's note and contractures in like 3 fingers. So it's very, very frustrating because I feel like the doctors just kind of... are also like guessing what to do with these patients and not managing them properly" P9

Medical management of hand injuries was largely inadequate. *Not enough doctor*s was a common complaint and *referral systems were inadequate*, with different challenges to referral being identified in

each of the practice settings. *Orthopaedic services were limited*, *late presentation* of injuries was common and *secondary complications* were prolific.

	***Contraptions & Contractures* Code**	**Quote example**
	Inadequate medical management	"I had once a patient walk into my office with some DIY dynamic splint made by a doctor that's not even like an orthopaedic surgeon who did like a tendon repair bedside, and this patient had already like, gotten contractures and everything. And then the doctor like when, like I went to go speak to him about it because he had... what he had done is, I don't know what it is called but it's like those metal like rods with the padding underneath. He had put the brace on the patient's wrist with bandages and then had put like a syringe, like a needle that goes onto a syringe. Under the patient's nails, and then had like, attached it with an elastic to this metal rod and told the patient that he needs to exercise his fingers using this. And then the patient, like the wound also went septic because he was also I think it was like a broken bottle that he was stabbed with or something. So when the patient came to me as he walked in with this contraption, like very brief doctor's note and contractures and like 3 fingers. So it's very, very frustrating because I feel like the doctors just kind of... are also like guessing what to do with these patients and not managing them properly. And they also want to discharge patients from the hospital very very quickly. So we see so many infections." P9
	Not enough doctors (1.38)	"We only have two doctors working in our hospital, and then they're from the Congo. And so they don't have a great understanding of exactly what is OT scope. So, even though we've been advocating and trying to educate about what gets referred to us there are and a lot of things that kind of don't get referred to us and gets kind of slipped under... Yeah, that we miss, that gets missed." P5
	Inadequate referral (1.13)	"Not having adequate referral systems between clinics and hospitals" P?<br>"Lack of communication between surgeons and therapists (surgeons are in a hospital 3 hours away and they don't write notes in the patient file" P ?
	Lack of orthopaedic services (1.38)	"And then we do have an orthopaedic specialist that comes to the hospital once a week, but he... I think he has COVID. So he's not been in a while "P9
	Late presentation (1.63)	"But yeah, we see a lot of really really bad scarring and a lot of complications. So there's a flexor tendon injury but then there's also nerve involvement. There's also hypertrophic scarring, the patient presents late, so I think this one I only saw like three or four months after he had his surgery" P1
	Secondary complications (1.75)	

Member checking:		
1.3 Contraptions & contractures	**No. of participants**	**Comments**
Yes, resonates	9	
Partially resonates	0	
No, does not resonate	0	

1.4 Insurmountable need

Participants were also submerged in an experience of insurmountable need. Two participants articulated aspects of this:

"The job makes me feel hopeless at times when I cannot help with the multitude of needs with my OT knowledge" P3

"Nobody in the hospital… can fix all these peoples' problems" P4

Having a *diverse caseload* contributed to this sense of need. Feelings of having *no idea* and being *eager and anxious* were common, as well as the sense of helplessness that came with being *unable to help*. Feeling *challenged, angry and frustrated* were common and link to being submerged in the complex context. The environment demanded *empathy* and participants commonly reported feeling *tired, emotionally exhausted*, and come felt at risk of *burnout*. Amidst these realities, participants agreed that they needed to be *able to prioritize needs*, *understand their own limitations* and have *emotional intelligence*. *Good time management* and *conscientious hard work* was demanded. Participants had to be *resilient* and able to *cope with stress, frustrations and challenges*. The realities also contributed to a need to *affirmed, heard and understood*. Participant's shared how they *tried to stay positive* and found *satisfaction* in *making small differences*.

	***Insurmountable need* Codes**	**Quote example**
	Eager and anxious	"She looks overwhelmed but eager to help with anything she can"P3
	Diverse caseload	"We see pretty much everything" P6
	No idea	"So, I had a lot of ideals and a lot of dreams. I was very excited to be here, but I didn't really understand what at all I was getting in myself in for. So I had all this big vision. I was excited to be in rural, but I really had no idea what it was about" P1
	Insurmountable need	"The job makes me feel hopeless at times when I cannot help with the multitude of needs with my OT knowledge" P3 "Nobody in the hospital… can fix all these peoples' problems" P4
	Challenging	"There are some cases that are very, very hard especially when it's assault... I had a lady whose boyfriend had beaten her up with an axe. So, sometimes it does it is quite challenging to deal with and kind of keep your cool in front of the patient so as to not like, "Oh my goodness, no!"" P9
	Frustrating	"So it's very frustrating that we can't then try and... this referral process that doctors get so stingy that they won't talk to us about it."P5
	Anger	"It's very, very frustrating, because I'm not someone that gets angry very, very easily, but I find myself especially when I'm seeing like X rays like this, or conditions, where there are contractures, developed where it could have been prevented like it is very very frustrating and I get very, very angry."P9
	Empathy (1.875)	
	Tired	"I feel exhausted" P5
	Emotional Exhaustion (1.13)	"She looks exhausted and overwhelmed" P5
	Burnout (1)	"I have almost burnt out and have since decided that I will only do my outlined duties and not volunteer to do all 8 of my colleagues' duties as well" P6

	Not able to help	"I feel like I'm... yeah there's so much going wrong and there's so little that you can actually do" P1
	Trying to stay positive	"She wishes she could always work with positivity" P3
	Making small differences	"She reminds me that the system will not be changed but you can change things where you are" P3
	Satisfying	"The job makes me feel humbled and satisfied"P9
	Prioritization of need (1.75)	Guidance from a supervising OT or mentor again would help with this. Management and community rehab modules from varsity helps here as well. P?
	Know how to prioritize (1.5)	
	Emotional intelligence (1.75)	
	Understand own limitations (1.5)	"Reflecting", "through gaining more experience", "Support groups with other comserv sharing their limitations"
	Coping with stress (1.625)	
	Coping with challenges & frustrations	"It works well to have it twice a month because you need that support so much. If it is less it might feel like you have suppressed all the emotions and challenges already and then you might not share them"P?
	Resilience (2)	"I'd Call Her Strong creative and kind"P8
	Heard and understood (1.125)	"... Even though you live in different provinces you feel so connected and heard"
	Affirmation (1.625)	"Through the group and my colleagues. However, if I could receive this from a supervisor, I feel like that would have been beneficial as well"
	Good time management (1.75)	
	Hardworking (2)	
	Conscientious	"Like you have integrity in yourself to do the thorough research on each patient...sometimes I think I wing it too much, to be honest. And I think I need to put more effort into it"P2

Member checking:		
1.4 Insurmountable need	**No. of participants**	**Comments**
Yes, resonates	9	"Lots of traumatic hand injuries" P9<br>"To survive you need to celebrate the small gains"P3
Partially resonates	0	
No, does not resonate	0	

1.5 Connecting through diversity

Another feature of the context in which participants were submerged, was diversity.

One participant reflected on the relative ease with which she had communicated with patients during university,

"Thinking back on patients I saw as a student, it amazes me like, we actually had a conversation whereas I haven't really had a full conversation with a patient this whole year. Some people can speak a little bit of broken English, but that's about it. So that really affects... even just the interpersonal relationship that you can build with a patient it's very limited which makes (one) feel quite isolated from one's patient, so it's difficult to connect."

Participants described many aspects in which their experience was *different from what I know*. One participant explained how her *prior exposure to diversity* through family life had prepared her for difference, but for others, *fitting in* was a challenge. Despite this difference, participants marvelled at the *sense of community* that they witnessed and saw the *beauty in the natural environment as well as the beauty of human participation* in everyday occupation. Participants expressed *gratitude* and a sense of having *grown* and gained invaluable *life experience/learning* through their CS experience.

As reflected in the quote, *communication challenges* were very common, placing demands on therapists to *converse in the patient's language*. *Cultural barriers* existed and necessitated an *understanding* and a *cultural humility* from participants. Mention was made of the need to respond well to *patient's traditional health beliefs and practices.*

	***Connecting through diversity* code**	**Quote example**
	Difference from what I know***	"...another life experience for me that's been, that's added to the whole treating hand injuries has been, come to the background I come from in terms of the socioeconomic status I come from and my upbringing versus the patients I am treating now, having time to make that mind shift change, of kind of what people use their hands for in these communities, also has a huge impact, because, you know, what I would be using my hands for, what my mom would be using her hands" P5
	Prior exposure to diversity	"So it was actually, so I would say my growing up and seeing all these different things made me more aware of how other people are living. So I think from my side that helps, because then I know that all my patients need to be able to make pap and needs to be able to go and fetch the water and be able to carry the heavy bucket because all of those things is a part of their lives" P8
	Fitting in	"She's like a fish out of water sometimes, however, can fit right in at other times as well" P7
	Cultural barrier	"Being white/English/afrikaans means that often there is a language and cultural barrier with both staff and patients" P9
	Cultural humility (1.5)	
	Cultural understanding (1.375)	"By taking the initiative and learning about others' beliefs and culture" P?
	Responding to traditional healing practices	"So often I've had patients come to me to say they are now injured, or they are now sick because they haven't accepted their calling to be a sangoma or to be a traditional healer... someone's cursed them"P5

	Communication difficulties (1.5)	"It's also difficult to chat to patients and like actually effort, like often I can't talk to patients at all. So, I rely on what I can see in my assessment. So I can see the physical problems, but I can't see like what they do at home, how they spend their time. So, then my assessment is not complete in terms of the occupational things. But I don't, I can't communicate with them"P1
	Therapist communicating in patient's language (1.875)	" The kit also needs to contain a resource that can assist the OT with speaking the patient's language, i.e. a dictionary or a link to an online course on the language"
	Basic competency in 2nd /3rd language	
	Sense of community	"It's different from what I know in that… many people do not have jobs, and is normal to sit around, but their sense of community is something I have never grown up knowing" P4
	Natural beauty (beauty of participation)	"The best of it has been helping those who need wheelchairs and the joy of them sitting happy and engaging in life" P8
	Gratitude (needs to move to insurmountable need)	"The job makes me feel appreciative of what I have"P8
	Growing	"She's like a flower struggling to grow through a mud patch" P3
	Learning & lifelong learning	"At the end of the year she'll… know more about life than 22 years could have tried to teach her and know that the hardships were worth it"P4

Member checking:		
1.5 Connecting through diversity	**No. of participants**	**Comments**
Yes, resonates	6	"Really enjoyed learning from my patients' cultures." P9<br>"Feeling "South-African", learning about culture and heritage and enriching my own view. P6
Partially resonates	3	• "(I) had to be reflective and make a concerted effort to be aware of my cultural blinders such that I can recognise the common and shared humanity amongst us, I like the term cultural humility that was used. I think the term connecting can be better substituted by learning or reflecting or thinking" P2<br>• "So often it was overcome, but I feel like at the end is some session I would sometimes feel like I wasn't able to do as much because of so barriers placed there because of diversity. But there was always the trying to overcome it" P7<br>• "I mainly said partially because a lot of the times I felt like, because the township was next to a city, was next to Joburg, the people were very… Westernised, if I can say it like that…I don't think it can really compare to culture in the rural areas…some people could speak English or even Afrikaans…the language barrier did affect me somewhat but not as much as the rural OTs and also there were always other people in the clinic who I could quickly go ask to translate for me…that was mainly the thing I didn't agree with. The others I agree with" P3
No, does not resonate	0	
Action: reporting needs to capture the variance and name of code needs to be changed		

Theme 2: Starting somewhere

This theme captures characteristics of the process or journey that participants experienced in delivering hand-injury care. The name of theme, is taken from *Bad Grad* who said that "everyone has to start somewhere". The five sub-themes are illustrated below (Figure 2).

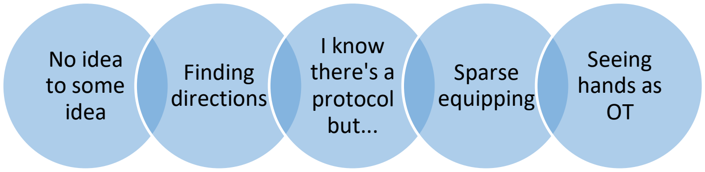

Figure 3: Five subthemes of Theme 2: Starting somewhere

2.1 No idea to some idea

The journey of delivering hand-injury care was often started with a sense of having *no idea*:

"So now I'm kind of stuck. I have no idea where to go with this hand. She is coming back sometime this week and then I need to do something. And I have no idea what to do" P5

A feeling or exclamation of, *'what it this?*' followed closely by desperate questions of *'what do I do?'* were common. Participants typically felt *overwhelmed* in the beginning, finding the task of hand-injury care *hard, daunting* or *scary*. Some reflecting on feeling as if they'd initially done *more harm than good*. A *lack of confidence*, a sense *of inexperience* and questioning their *competence* was common.

This sense of *no* idea was, however, not terminal: participants demonstrated that they were *willing and trying* and worked towards *finding their feet*. A gradual orientation towards *starting somewhere* was achieved. This came with a sense of *doing better than one thinks* and a sense of victory in being able to *do something*. Gradually they acclimatised to *thinking on their feet* and had experiences of *success* and a sense of *satisfaction* in delivering hand-injury care services.

	***No idea* code**	**Quote example**
	No idea	So now I'm kind of stuck. I have no idea where to go with this hand. She is coming back sometime this week and then I need to do something. And I have no idea what to do. P5
	What is this? (1.63)	Because I had a patient who came in and he had contractures, which made both his hands look almost like spider man hands, so you know when Spider-Man does his thing and the webs come out. His hand literally was stuck in that position. P8

	What do I do? (1.5)	I didn't know what to do! P5
	Overwhelmed (1.25)	Sometimes when you start seeing hand patients you get really overwhelmed and you feel like you don't know anything. P?
	Daunting	We didn't really have that much experience with hands in varsity, so I was actually very apprehensive to even start treating hands. I didn't feel comfortable, and I didn't feel, like everything else was already challenging as is and I had practised the treating strokes... but hands I had no experience with. So...it was a very daunting experience and...I didn't even know what it was about, to be quite honest. P4
	Scary	
	It's hard (1)	So it's I guess starting to come easier and, but it's still not easy I guess it's still stressful at times, and you kind of just wish you could just grab the right splinting material and make the right splint and that you need to make. P5
	In experienced (1.75)	We are so inexperienced, specifically in HT after uni and we need the support. P?
	Lacked confidence	I didn't know what to do. I didn't feel confident in my competence, I didn't feel competent enough when it came to hands. P5
	Question of competence	
	More harm than good	Like my splints in the beginning of the year probably did more damage than they did good. P2
	Willing and trying	Alot of times with hands that I do something and I think it will work, and then it doesn't, and then it's kind of that awkward situation where you have to explain, you're inexperienced and they just have to bear with you, you're trying the best you can. P3
	Finding my feet	At the beginning...I was finding my feet . P2
	Starting somewhere	She reminds me… everyone has to start somewhere. P4
	Doing better than one thinks	What she doesn't know is that she is doing well, as she provides more for patients than they expect. P6
	I can actually do something	Realising that actually I have grown. I don't freak out. I know how to assess even when I don't know what's going on, I can actually do something. P1
	Improving / succeeding	I think my performance as a therapist in terms of hands has definitely improved and what I'm able to give patients. P8
	Satisfying	So this patient, I wanted to share because it was like a little bit of a success story. P9
	Thinking on your feet	It's been 6 months and I'm getting used to having to think on my feet and problem solve. P5

Member checking:		
2.1 No idea to some idea	**No. of participants**	**Comments**
Yes, resonates	7	
Partially resonates	2	"I think sometimes we had an idea to start with – like doing our basics, doing assessments and seeing what they can and can't do and then going from there" P8<br>"I like no idea more…When we're in a panic mode, we tend to become very black and white in our thinking and so as soon as we're put up with a situation, it's like fight, flight or freeze mode. And so you go very much into, "Oh my word I know nothing!". Or you run away. Or you're

		like, "Yes – come on – let's do it!". And so I think it's a gradual kind of process of, "Oh my word I know nothing!" But there's a patient here and you need to do something. And I just always heard my lecturers saying in the back of my head, "You know more than you think"… And just recognising that I wouldn't be there if I knew nothing. So that's why I say 'some idea'. I don't have the whole idea. But some idea." P2
No, does not resonate	0	
Action: this sub-theme was presented to participants as "No idea" although I suggested in the member checking session that that 'no-idea to some-idea' was an alternative. These comments highlight that this change to the name of the sub-theme was a necessary change to make.		

2.2 Finding directions

This sub-theme captured the challenge of clinically reasoning around hand injuries as an inexperienced therapist being *Submerged* in the contextual realities outlined in *Theme 1*. The Anxi-cited Outsider explained:

"I obviously didn't know much about hands at all, but I am an avid Googler, so whenever something is there, I am not shy of taking out my phone and looking up what or how or why. But there's also this thing about the lack of diagnosis." P6

This quote captures both the challenge of *conditions new to me* as an inexperienced therapist, but also the compounded challenge of *finding directions without diagnoses*. Delivering hand injury care demanded that participants clinically reason around chronic conditions, reason around injuries, particularly acute and traumatic injuries. They were frequently required to treat patients with fractures, upper limb impairment post-stroke and patients with burns to the hand.

These demands were accompanied by a desire for *learning opportunities*, a need to *learn-by doing* as well as a to *share their knowledge and experience* with others in similar contexts. They reported a need for *knowledge and skill*, along with *support for developing basic hand therapy skills*. They frequently sought *guidance* to aid their clinical reasoning, and *feedback* to consolidate and learn from their actions.

	***Finding directions* code**	**Quote example**
	Conditions new to me	When like seeing this diagnosis and then seeing the presentation, you kind of feel a bit confused, like I was like I have never heard of this, I don't actually know like what the prognosis is. I actually don't know what the medical treatment of this is, so I had to go read up a lot on it. P9
	Finding directions without diagnoses (1.63)	I obviously didn't know much about hands at all, but I am an avid Googler, so whenever something is there, I am not shy of taking out my phone and looking up what or how or why. But there's also this thing about the lack of diagnosis. P6
	Reasoning around chronic conditions	But we did have quite a lot of arthritis patients and patients with a lot of almost... a lot of hand pain. So I'm not too sure what the pain was what it indicates or what the pain really meant. P8
	Reasoning around injuries (1.71)	So when I see people with still open wounds and people have been like had trauma to the hand. I can see that I don't really have any of that knowledge. P8
	Treating patients with acute/traumatic injuries	Yeah. So, there's quite a variety, but, yeah, about 20% of that is hands and mostly stab wounds or fractures. P3

	Managing burnt hand	I was wondering if you could help... I have this patient...who sustained burns to her hand ... I'm lost now because I don't know how to correct the contractures if her skin can't yet handle the pressure? P5
	Managing stroke UL	Alot of hands that I treat are stroke injuries. P3
	Managing fractures	I don't have a specific case, but can we please talk about radial, supracondylar and humeral fractures, especially the stiffness after removal of POP? P6
	Knowledge (1.625)	I am the only therapist and at times would like to have someone with a bit more knowledge to guide me. P?
	Skill (1.625)	Online courses focus on theory and not clinical skills thus it is difficult to gain the skills. P?
	Guidance (1.625)	Having that community who can guide you when you are struggling with your hands patients. P?
	Feedback	And especially with something that's so like hands-on with hands you know you're making the splints, you're doing the... I remember scar massage and only like three weeks ago the supervisor told me no, I'm not massaging with the pressure that I'm supposed to be massaging. And you know those things I just never knew because I never did before. P2
	Skills support	
	Opportunity to learn (1.75)	There are limited courses available to learn more about hand therapy. Or timing of the courses/ cost becomes an issue. The group however has aided in my learning this year a lot. P?
	Share experiences & knowledge (1.375)	I would also really appreciate the opportunity of being able to discuss my and other people's hand therapy cases with other therapists and be able to problem solve and discuss cases together. P1
	Learn-by-doing (1.625)	By having patients attend therapy I have to learn by doing as I have to read up on the condition and then do it immediately. P?
	DG decision making	As a commserve OT you get so many referrals for DG assessments and because you are inexperienced it is something scary. It worked so well that she outlined the eligibility criteria. P?

Member checking:		
2.2 Finding directions	**No. of participants**	**Comments**
Yes, resonates	9	"Agree a lot with guidance" P7<br>"Often googled and paged through my university hands book" P9
Partially resonates	0	
No, does not resonate	0	

2.3 I know there's a protocol but…

This sub-theme communicates the unorthodox presentations and the more advanced demands that this places on the therapist's reasoning due to 'curveballs' or complications associated with contextual features (illustrated in the first theme):

"And then when a hand injury will come in that doesn't suit a certain box or like a certain picture." P5

With protocols usually outlining typical presentations and timeframes, these were less useful when patients presented long after surgery or were never able to access surgery. There were numerous *challenges to follow-up* appointments with patients and clinic outreach demanded mobile or *travelling hand therapy* services. *Patient adherence* was often a problematic demanding *appropriate intervention guidelines, education and home programmes* that were responsive to these realities.

	I know there's a protocol code	Quote example
	I know there's a protocol but... (1.88)	And then when a hand injury will come in that doesn't suit a certain box or like a certain picture. P5
	Travelling hand therapy (0.88)	Being able to transport all equipment easily has been a task that I find difficult and frustrating
	Challenges to follow-up	Patients cannot follow up with therapy due to cost of transport, accessibility to transport and often the hospitals are very far from their homes. (all)
	Patient adherence	Research must be done on patients to learn their perspective on home programmes and what can be done to improve compliance to these home programmes. Guidelines to adhere to can be published in a leaflet for OTs to make use of in rural communities, as many patients can only be seen once a month and home programmes are vital. (all)
	Appropriate education & home programmes	
	Appropriate intervention guidelines	The kit should contain a resource that details the most common hand injuries treatment guidelines that can be passed on to the next novice therapist. (all)

Member checking:		
2.3 I know there's a protocol but…	**No. of participants**	**Comments**
Yes, resonates	9	"Seeing pain and contractures without diagnosis and adding a language barrier" P9
Partially resonates	0	
No, does not resonate	0	

2.4 Sparse equipping

Participants' journey of delivering hand-injury care was further characterised by sparse equipping on many fronts. *Dedicately Winging-it* explains some of these:

"I am the only OT in my department or… we're a Community Health Clinic there's no permanent staff... since 2017 in rehab... It's quite hectic especially because ... it's quite a busy clinic..., I remember the first few weeks, was the most overwhelming thing I've ever experienced... especially with hands...(I) went from having absolutely no clinical experience (at university) ... And now I'm like here with this three-year-old child that's just his fingers burned, and now I must do stuff. And it felt so unethical and I'd like run around trying to call my supervisor who's like in (a district) hospital and she obviously also trying to be a therapist there, and finding out what I must do. "P2

As evidenced in this quote*, undergraduate preparation* was considered by some to be inadequate with a *lack of practical or fieldwork experience* in hands being frequently highlighted. Supervision was often absent or unsatisfactory. While therapists with supervision were grateful for this, it was identified that this could be both an *opportunity (for learning and support) but also a barrier* to feeling free to explore therapy without a sense of being watched. Practice conditions were largely accompanied by a need for *support* and a need for *mentoring and supervision*. *Reflection* as a means to 'learning from yourself' was considered to be vital, demanding the necessary *reflective practice skills* to achieve this.

Being *the only OT* was challenging for a number of participants who found themselves in this situation. Unsurprisingly, many expressed a need *for colleagues*, to feel *not alone* and for the creation of *OT posts*.

Courses were often not well suited to need, highlighting a gap for *appropriate CPD opportunities*. Circumstance also called for therapists to know *how to search for evidence*.

Limited resources and *inadequate hand therapy equipment* were common. Although this provided an opportunity to nurture their *resourcefulness and creativity*, the need for resources was clear: *appropriate splinting equipment, appropriate consumables* (eg. splinting material), *versatile resources that could be used for a diverse generalist caseload*, and *appropriate assessment and treatment resources*. Participants articulated a need for an *organized treatment station* that was *portable and ergonomic*.

	Sparse equipping codes	Quote example
	Practical undergraduate exposure	And then a positive would be that I spent a (fieldwork) block at (Academic Hospital)... so the whole Thursday we would sit in the hands department, and we would deal with all the walk-ins... I had a very good experience there just because I got to see so many different hands and then it was helpful to have someone who was always supervising and come to check what am I doing, what is this and what protocols to use. So, I would say those were some life experiences that contributed to my hands experience. P8
	Sub-optimal undergraduate prep(1.57)	So that experience (doing splints at varsity) really just made me feel like that my best really wasn't enough, like not even to pass, those type of things. So, it made me feel very incompetent and like not really enthusiastic about hands. P8
	Limited resources	So with this picture, I wanted to show that there are very little resources in terms of hand therapy. On the left side, there's blocks that you usually put into a little, those balls that has holes with different shapes you put the blocks in there. It's kind of a kid's toy so it's always difficult to explain to the patient to just bear with me. I know, it's silly, but it's all we have. Shame and they're usually quite accommodating. P3
	Inadequate equipment	But my splinting bath is also one of those cooking, pans, which is very frustrating because the lowest temperature is too hot. So, if you're distracted by cutting velcro or something, your splinting material can melt too much, and then it's just like a sticky, a sticky mess. P9
	Inadequate supervision	I am the only OT in my department. Or it's only comserves because we're a Community Health Clinic there's no permanent staff... since 2017 in rehab... It's quite hectic especially because ... it's quite a busy clinic..., I remember the first few weeks, was the most overwhelming thing I've ever experienced... especially with hands...(I) went from having absolutely no clinical experience (at university) ... And now I'm like here with this three-year-old child that's just his fingers burned, and now I must do stuff. And it felt so unethical and I'd like run around trying to call my supervisor who's like in (a district) hospital and she obviously also trying to be a therapist there, and finding out what I must do. P2
	Supervision as opportunity & barrier	I've been very fortunate because I've had somebody kind of guiding me through the entire process and it's quite nice to be able to have somebody there just to like double-check yourself and it kind of makes you a bit more confident in what you're doing. So, I'm very fortunate that way but in the same breath, it doesn't allow you the opportunity to kind of explore and do things yourself. So I think there's a bit of good and bad in both aspects. P4
	Courses not well suited to need	Online courses focus on theory and not clinical skills thus it is difficult to gain the skills
	Appropriate CPD	I managed to find like a really comprehensive hand therapy course which I've been doing and like I've really been enjoying it. P1
	Support (1.5)	For me, a huge thing that I had to do was I had to start debriefing with someone. And so that really helped me a lot. So I started seeing a psychologist on a weekly basis... I tended to take a lot of my work home with me. P5
	Mentoring & supervision	Find a mentor/supervisor who you can discuss cases with and help grow your clinical reasoning. Think outside the box when it comes to therapy in order to actively engage patients and see results. P?

	Reflection (1.5)	I have learnt what to do and what not to do through this year, by reflecting on my cases I have had through the year. Seeing what has worked, is working and what is not working. Also learning when to ask for help. P?<br>Do a lot of reflection you learn so much from yourself as well. P?
	Reflective practice skills (1.87)	
	How to search for evidence	Need to know how to find literature, do research and network with experts and other therapists so that you can teach yourself and learn when you don't know what to do. P?
	Resourcefulness /creativity (2)	I think originally it was quite stressful, but the more I've had to do it, the less stressful it's become, I guess because I'm kind of getting more used to having to think on my feet and just use what I have around me. P5
	Sparse equipping code	Quote example
	The only OT	The OT department makes me think of a one man sailing boat with some holes and buckets to keep the water out and duct tape on leaking parts
	Not alone (1)	Since I am the only OT it is hard for my colleagues to assist me as they are from different professions
	For Colleagues (1.25)	I am the only therapist and at times would like to have someone with a bit more knowledge to guide me
	OT Posts	I have a fellow comserve Physio but it would have been great to have an OT. Can be met with more permanent OT posts in government.
	Resources	(in answer to the question what other needs?) Mostly resources
	Appropriate splinting equipment	OTs were sometimes lucky to have a splinting pan, however, if it were round, it resulted in the splinting material not being able to fit into water.
	Appropriate hand therapy consumables	At the beginning of the year, I was given one small sheet of splinting material, only one type of thickness and a small amount of soft material. Now we are out of material for both soft and thermoplastic splinting and I have been making a plan with POP.
	Versatile resources for generalist caseload	A variety of resources to treat not only hands patients
	Organized treatment station	At my hospital we don’t have a lot of resources but we do have a decent amount. The problem is that there is no storage space so then everything is on the one little table in the treatment room and it becomes very cluttered and disorganized
	Assessment and treatment resources	Including both assessment and treatment tools that are easy/light to transport and are versatile (can be used for more than one goal/aim).
	Portable, ergonomic hand therapy station	Thus, the kit has to be well resourced to assist quick and effective solutions to assessment and treatment and it must be able to be taken to patients. It must be compact and easy to use so that it saves time between the short sessions.

Member checking:		
2.4 Sparse equipping	**No. of participants**	**Comments**
Yes, resonates	9	"More undergrad focus on hands and OTs amazing role in it" P3<br>"Undergrad experience was so minimal for me so I really resonate with that" P5<br>"Expired materials, Complacent permanent OT's" P6
Partially resonates	0	
No, does not resonate	0	

2.5 Seeing hands as OT

This sub-theme captures another dimension of participants' journey: the consolidation of their appreciation of occupational therapy for patients with hand injuries. Some participants started the journey seeing a disjuncture between hands and OT:

"So, I guess as a student I kind of felt that hands was kind of more physio-ish. I didn't really see it as being that OT." P1

For the latter therapist and one or two others, they initially experienced a *dislike or disinterest in hands*, holding the impression that hands was *not very OT*. However, their journey of hand-injury care in CS was integral to their learning *to see hands as OT* and *appreciating the link between the hand and occupation.*

Participants endeavoured to *understand what people use their hands for*. In rural contexts, this meant understanding and being able to analyse unfamiliar occupations, like cindering a floor (a process of sealing and polishing the floor of a hut using a mixture of cow dung and mud. The occupation is typically performed in four-point kneeling).

This learning was accompanied by a need to *figure out activity and occupation* and to deliver therapy that was *occupation-based*. This demanded *resources for occupation-based hand therapy* and a desire for *activity ideas* to relieve participants from experiencing that they were doing the same thing with every patient.

	***Seeing hands as OT* code**	**Quote example**
	Dislike or disinterest in hands	I also did not like hands at all coming into conserve. P5
	Not very OT	So, I guess as a student I kind of felt that hands was kind of more physio-ish. I didn't really see it as being that OT. P1
	Learning to see hands as OT	I think I saw maybe two hands patients throughout my four years of prac. So, I didn't really ever get that exposure to see how, I mean, it's obvious now, but again as a student I didn't really see how completely integral to occupation the hand is and how you can't do occupation without the hand. P1
	Appreciating the link between	

	hand & occupation	
	Understanding what people use their hands for	You said something now about living yourself into this person's occupation. Today I mock chopped wood with my patient. So, we stood there whether it's fake or not, not actual like invisible axes in our hands and just like did this chopping motion. I'm sure that if anyone was looking at us, they would find it very, very hilarious. So that's something that I often do with patients. I try to live myself into their occupations, their daily life and to really figure out what do they do, what do they want to be able to do and what did they do before. P6
	Figuring out activity and occupation	I experienced exactly the same thing... I find it so frustrating because I have the same experience because like often the patients will come to me and they'll say, you know, their biggest concern is for example washing their clothes and then I'm like okay, but now they, I can't, what do I do with them? I can't bring out a bucket of water and soap and we sit here and hand wash clothes or, you know, their issue is sweeping their yard or, you know, such cultural based activities that you almost can't replicate in the hospital setting, which I find very hard to replicate. And then therefore I also just become solely exercise based, like I can't actually think of a time that I did a kind of occupation based activity with the hands patient just because I find it so hard to implement or like, not implement, to recreate an authentic kind of experience. P 5
	Occupation-based therapy	Sometimes when you start seeing hand patients you get really overwhelmed and you feel like you don't know anything but don't forget what you learned at varsity. You do know the basics. You might have to brush up on some of the technical skills and knowledge but don't let that overshadow what you already know about occupations! Don't be afraid to brainstorm and experiment with your patients in terms of finding new ways of doing things but make sure that you're always focusing on the occupation. Don't lose your identity as an OT while you're trying to improve on your biomechanical knowledge and skills. P?
	Activity ideas (1.75)	the actual treatment activities because that's also something I struggle with. Also ... not having a lot of resources like I don't have the ability to make a sandwich or make a cup of coffee or whatever with my hand's patients. So often I feel like just doing the same things over and over again. P5
	Appropriate OBHT resources	It is important to do occupation based activities for example handing washing on a washing line and securing the clothes with pegs. The kit should have materials that can work for a diverse range of fine motor skills.

Member checking:		
2.5 Seeing hands as OT	**No. of participants**	**Comments**
Yes, resonates	7	
Partially resonates	2	"Personally didn't have to learn to like hand therapy and see it as OT, but did learn to love it more" P7*<br><br>"I didn't agree with it because I always plainly saw hands as being OT so I didn't really feel like it's a physio thing… I always tried to make it occupation-based… of course with limited resources and not having a lot of things you can do occupation-based therapy with, it ended up being more physio-like… so exercises…At varsity I made that link." P3*
No, does not resonate	0	
Action: the comments made it clear that in describing the theme, variance needs to be captured. The sub-theme requires a name revision in order to accurately capture this.		

Theme 3: 'Dynamics of surthrival'

The third and final theme sought to capture dynamics that either supported or enabled growth and learning, and a strong response to the demand for hand-injury care, or dynamics that hindered this. The theme resonated strongly with participants, one of which explained it as "the transition between thriving and just surviving". Appreciating that the experience of aspects of 'just surviving' and 'thriving'

were dynamic, the theme captures thriving and survival on a continuum with various factors, processed and experiences influencing movement along the continuum.

3.1 Propelled by demand

The first sub-theme interpreted the *diverse caseload* that participants had as pre-cursor or antecedent of growth. The demand for hand-injury care forced them to learn. Their experience of engaging in the task of hand-injury care was, however, a dynamic experience that was sometimes accompanied by more or less of an experience of thriving or surviving. *Illusionless Optimist expressed this:*

"I was just thrown in the deep end and I was the only OT and I had to somewhat drown. Which has been negative in some ways because like there's no-one to check if you are doing things right, if you stuck, then you're stuck. But then I think it also has been in other ways positive because it's forced me to learn and read up and like really investigate hand injuries" P1

	***Propelled by demand* codes**	**Quote example**
	Complex diverse caseload	I would have liked a more stimulating environment for learning or a more acute setting<br>Vs Many hand injury patients at the hospital
	Forced to learn	I was just thrown in the deep end and I was the only OT and I had to somewhat drown. Which has been negative in some ways because like there's no-one to check if you are doing things right, if you stuck, then you're stuck. But then I think it also has been in other ways positive because it's forced me to learn and read up and like really investigate hand injuries
	Dynamic	I think at the beginning I did mine separate because I was finding my feet and trying to understand, you how, what is even P and E and O for myself. And now and then I drew it a lot more closer together and then I stopped and I thought and I was like, but actually I don't know if that's completely accurate. I think that there are days, there are times when it's like that and then there are times when it's like very congruent and then there's times where they are just touching. I said that. It's like a dynamic movement between those things. It's not where it was in January, but it's definitely not always completely in sync.

Member checking:		
3.1 Propelled by demand	**No. of participants**	**Comments**
Yes, resonates	9	"Thanks Google" P6<br>"Was forced to learn for sure" P9
Partially resonates	0	
No, does not resonate	0	

3.2 Agentic Momentum

An additional propelling force was the sense of agency exercised by participants to respond to the demand for hand-injury care. Participants were aware of their responsibility to deliver quality care and actively sought ways to enable this. Voluntarily joining the CoP was one evidence of this, with accessing expertise from a referral facility being another:

"So what I actually did was I did go to (a regional hospital), I asked to join them for almost like a job shadowing, for two days just doing their hands OT. Just because in the beginning I was very overwhelmed. With how many hands patients and feeling very inexperienced" P8

Further evidence of agency was seen in them *taking initiative, immersing themselves in their context, researching,* and *reflecting.* They *established support networks, sought support and expertise, made attempts to cross language barriers* and *took to learning-by-doing*. They negotiated the need for reasoning around hand injuries through *problem-solving*, coaching themselves to go *back-to-basic* and *thinking on their feet*. When unsure of how to proceed with a patient, they centred themselves *by focusing on clients' participation and function*. *Identifying their own support and development needs* was further evidence of their exercising agency.

	***Agentic momentum* codes**	**Quote example**
	Taking initiative	By taking the initiative and learning about others beliefs and culture
	Immersing self in context	By attempting to immerse yourself in the culture of the institution
	Researching	But then I think it also has been in other ways positive because it's forced me to learn and read up and like really investigate hand injuries whereas hands wasn't really something that I was particularly interested in, but because I felt so lost in it, I had to do a lot of research and I have made amazing connections and got to know so many amazing people who've helped me so much. P1<br>I think I could have read, to be honest... I could have read up a lot more than I did. P2
	Reflection	Do a lot of reflection you learn so much from yourself as well. P?<br>Also, a hand therapy specific reflective practice template can be included in the kit to help novice therapists reflect better and thus have an active learning approach to their own therapy. P?
	Seeking support & expertise	So what I actually did was I did go to (a regional hospital), I asked to join them for almost like a job shadowing, for two days just doing their hands OT. Just because in the beginning I was very overwhelmed. With how many hands patients and feeling very inexperienced. P8
	Establishing support networks	Through the hands CoP, groups on WhatsApp of OTs in final year class, specific interest groups of rehab/allied commserves, where people ask questions/for help. P?
	Attempts to cross language barrier	I understand most of my patients and make an effort to speak in their home language. P?
	Learning by doing (1.625)	Lots of opportunities at work. P?
	Back to basics	Sometimes when you start seeing hand patients you get really overwhelmed and you feel like you don't know anything but don't forget what you learned at varsity. You do know the basics. You might have to brush up on some of the technical skills and knowledge but don't let that overshadow what you already know about occupations! P?
	Problem solving	I didn't have the right splinting material so... I had scraps of the 3.2-millimeter materials so I made the splint out of the 1.6 millimeters and then tried to reinforce it with like strips on the thicker material. P5
	Think on your feet (2)	I think originally it was quite stressful, but the more I've had to do it, the less stressful it's become, I guess because I'm kind of getting more used to having to think on my feet and just use what I have around me. P5
	Focus on client's function & participation	Even after years of people not being diagnosed, and then they come to me and then it's kind of only the adaptations to left over. I'm quite logical and I like to think of new ways for a person to do something. P6
	Identifying own support and development needs	**There's** still a lot of room for growth, but I think I can kind of, I think it's a thing of conscious incompetence, where now I can kind of plot the way forward. I can see I need growth in this and this and this area, whereas in January it was just like I don't know what I'm doing.

Member checking:		
3.2 Agentic momentum	**No. of participants**	**Comments**
Yes, resonates	9	"I really liked this theme! I think it's really important" P1<br>"Especially taking it upon myself to learn a new language" P5<br>"Didn't think about it like this :)… agree – love this "P9
Partially resonates	0	
No, does not resonate	0	

3.3 Support for learning

This sub-theme communicates the supports that enabled growth and supported a shift towards thriving. As comments from member checking indicate, it is necessary to acknowledge the possibility of the inverse when supports were not in place (see comments in member checking box).

As evidenced by this quote*, case discussions* and *support for clinical reasoning* were experienced as helpful:

"The most valuable aspects of her session was talking through cases and getting advice on how to approach them. This helped to grow my clinical reasoning as well as provide mentorship that was desperately needed" P?

In addition, *undergraduate preparation, reflecting and discussing with colleagues, appropriate courses, social media* and *relationships with doctors* were identified as additional supports for learning in hand-injury care.

	***Support for learning* codes**	**Quote example**
	Undergraduate preparation	I had a good relationship with hands to begin with, because although like the splinting made me not feel so great about hands, doing a block at (Academic Hospital) for seven weeks. P8
	Cognitive informational support	Previous university notes, research and the whatsapp group
	Support for clinical reasoning	The most vaulable aspects of her session was talking through cases and getting advice on how to approach them. This helped to grow my clinical reasoning as well as provide mentorship that was desperately needed.<br>As CSOT you really come in knowing. Sometimes I felt guilty about how little I knew and how bad my therapy was for my patients so being in a group with other people who are in the same boat as me was so helpful. But then also being given access to people who could actually help me and teach me how to clinically reason through cases felt like winning the lottery in terms of hand therapy.
	Case discussions	I would also really appreciate the opportunity of being able to discuss my and other people's hand therapy cases with other therapists and be able to problem solve and discuss cases together. P1
	Relationships with doctors	(The orthopaedic surgeons) quite respect the OT profession as well which is quite nice. They definitely respect like where we come up, how beneficial splinting can be and mobilization, and all of that stuff. So it's really nice to be respected in that sense. P4

	Reflecting & discussing with colleagues	Talking to other conserves also in rural or periurban areas helps to form an understanding of our client population. Can be met more with specific city or Provincial discussion groups for rehab staff. P?
	Appropriate courses	I managed to find like a really comprehensive hand therapy course which I've been doing and like I've really been enjoying it. P1
	Social media	Can be met through groups on Facebook as well where people share ideas.

Member checking:		
3.3 Support for learning	**No. of participants**	**Comments**
Yes, resonates	7	
Partially resonates	2	• "The CoP did the most of this for me ... the rest not so much…I felt like I got the most support for learning more about hands and knowing what to do through the CoP and the Whatsapp group that we had. Not so much the appropriate courses – I tried one course which I just felt was not appropriate at all for rural hand therapy. It was very intense, so I didn't really find that that helped me a lot. In terms of under, we, if I'm being totally honest, we had a really poor lecturer and I really struggled to grasp the concept of hands in undergrad because everything was kind of taught on videos and Youtube videos and nothing was really practically taught and explained. So I think that's what I meant by that…and that if those other aspects, such as, if there was kind of an appropriate rural hand therapy course, like you kind of mentioned in the feedback, where they were looking at 'there's a protocol but not so specific to first world hand therapy where Day 1 you do this, Day 4 you do this.. whatever…that would have be been very helpful. Better undergrad prep would have been so so helpful for me. And then…Ja… if I'd had better doctors at the hospital that understood hands…would've also been great…I definitely agree that if I had…if those other aspects were available to me it would have supported me in thriving more in hands than just surviving, cause I did feel with hands that I was just surviving through Comserv with a little bit of knowledge and Google that I had available" P5<br>• "Needed for surthrival, limited in CS and undergrad…Support for learning is such a big necessity for, thriving, especially when you are a newly graduated OT. It felt like undergrad taught me a lot of things yes, but I was not nearly prepared enough to start working, especially not in rural. I would also have benefitted more and thrived more, if a close mentor (in the same hospital, with the same patients) was able to discuss and reflect on cases in a valuable way. There I think the "culture of complacency" played a big role, as many of my supervisors just depended on me, the CS, to do all of the work" P6
No, does not resonate	0	

3.4 Resources

Similarly, *resources* and *up-referral resources* supported learning:

I referred (the patient to the regional hospital) just because I honestly didn't know what to do with them. P8

	***Resources* codes**	**Quote example**
	Resources	Many hand rx modalities available at my hospital.
	Up-referral resources	I referred (the patient to the regional hospital) just because I honestly didn't know what to do with them. P8

Member checking:		
3.4 Resources	**No. of participants**	**Comments**
Yes, resonates	6	
Partially resonates	2	"I did not really agree with this because I didn't really have the same experience in terms of referral. Often hands patients would be kept at our hospital even if it was for surgical intervention, they would rarely be referred to a different hospital and I didn't have many…many um…treatment modalities or anything like that and I also … for some reason I remember P6 saying that there were other therapists and things that she could learn from as well which…I was mostly by myself…so I think that is why I put "sometimes". P9<br>"Was not able to refer as I was the up referral"
No, does not resonate	1	"I feel like I was completely on my own and didn't really have many resources" P5 *
These comments indicate that communication of the sub-theme needs to make clear that the presence of resources supports thriving, and the absence of them does the inverse.		

3.5 Emotional supports

The need for emotional supports is captured in this sub-theme.

"I am able to go home on weekends and see my pets and my family"

Participants shared that becoming *desensitized* or *dissociating* were common protective emotional responses with some participants using *debriefing* and the *setting of boundaries* to cope emotionally. *Emotional support* was largely accessed through *family and friends*, *colleagues and other community service OTs*. The affirmation received through *patient feedback* also acted as an emotional resource. Some participants indicated *that staying home, or being able to visit home* was a support. One participant felt that if she had been able to stay home and access her usual supports, this would have been an enabler.

	***Emotional supports* codes**	**Quote example**
	Dissociated or desensitized	So sometimes it's very very gross... other times, I ...I think you get a bit desensitized. P9<br>I like dissociated a bit in a sense and I was just kind of like, do what you kind of think, and don't think too much about it, otherwise it's going to be overwhelming. And like you're going to feel really bad about yourself, and so you stopped caring to a certain extent and you just kept going. P2

	Setting boundaries	...Having that clinical boundary... like learning to have that professional, clinical persona. Being able to know the difference between kind of when to be empathetic and sympathetic, I guess. Learning that kind of difference. P5
	Debriefing	My supervisors have been very empathetic towards me and… but also, they shared in that anger that I felt. Especially in the last week, I had a very, very sad case. And when I got back to the offices I was able to tell them about what happened and they shared in my anger and they shared in my hurt. So my supervisors are very nice in that regard... definitely, debriefing. P6
	Emotional support	There are people at my workplace that are very supportive. However, it would have been beneficial if I was able to stay at home where my family, who is normally a large support system for me, is. Including my partner and friends. P?
	Friends & family	Family and friends listen and support. P?
	(Sources of support)	Family, friends, fellow comm serves. P?<br>I am able to go home on weekends and see my pets and my family P?

Member checking:		
3.5 Emotional supports	**No. of participants**	**Comments**
Yes, resonates	8 (9)	"needed a psychologist to make it through the year" P5<br>"YES" P6<br>"This was an important factor for me. Saw lots of abuse/violent crimes etc" P9
Partially resonates	(1)	(Desired support in the workplace ito guidance P8)
No, does not resonate	0	

3.6 Catalysts

Immersed in the data over an extended period made apparent to the researcher that there were certain factors that 'hit-the-spot' in terms of participants needs and acted as catalysts for moving away from only surviving. One catalyst, the presence of immediate or *real-time support* is evidenced in this quote:

"The WhatsApp group where Kirsty would answer questions "in real time" (i.e. while you're sitting with your patient in the session)" P?

Although peer support was used, having *access to expertise* was considered invaluable. The *proximity of support* mattered: in-situ support was desired. *Support that specific or responsive* to immerging needs was also valued over generic input or teaching. The *CoP* as a mode of learning and support also proved to be catalyst for thriving due to its versatility in meeting many of the needs articulated by participants.

	***Catalysts* codes**	**Quote example**
	Timing of support / 'real-time'support	"The WhatsApp group where Kirsty would answer questions "in real time" (i.e. while you're sitting with your patient in the session)" P?<br>I found this group extremely helpful - It allowed "instant" sharing of ideas, receiving advice and mentorship. P?
	Proximity of support	It was in the beginning when I worked a lot with the other OT at the hospital.
	Specificity of support	She was also one of the sessions we asked for specifically which I think is important for the hands COP - identifying what the members needs are and then responding to that, as was done. So it's not just another lecture or course but it is specific.

	Access to expertise	The experts also helped a lot with this - being able to take questions to an expert you would maybe never have had access to - was so amazing
	Community of Practice	Quote:<br>This need (for **knowledge**) is being met through the community of practice<br>The group has also assist in showing how to do certain therapy **skill**<br>The COP allows for this **reflection**<br>Whatsapp group provides **guidance**<br>Through the hands CoP it helps to have **feedback** that what you wanted to do was right<br>Hands CoP has been helping to facilitate this **learning by doing.**<br>The COP has been great in allowing the platform to **share experiences**<br>Also mentioned as source of support, activity ideas, means to being "not alone", meets need for colleagues<br>"I can't think of any other needs. However, I just wanted to say that the hands group this year has been a huge help with meeting my needs of the year, and I think going forward, if a group like this, for other community service OTs can happen, it would be of great benefit to them"

Member checking:		
3.6 Catalysts	**No. of participants**	**Comments**
Yes, resonates	9	"100% agree!" P1<br>"Access to mentorship programmes and real time support/COP is a wonderful benefit! Whatapp groups between graduates is also an idea" P3<br>"real time support!!" P9
Partially resonates	0	
No, does not resonate	0	

Phase 5: Refining, defining and naming themes

Still to complete, although some overlap of Phase 4 and Phase 5 has occurred.

Phase 6: Writing the report

Still to complete

Identifying support and development needs (second part of research question)

In order to extrapolate support and development needs, the experience captured in each sub-theme was considered/interrogated with the following questions:

What support and development needs emanate from this experience? (Those met, those not met). What would make it easier for CSOTs to deliver hand-injury care?

The list created for each theme broadly fell into the following categories:

- Service / system
- Opportunities for CSOTs to develop knowledge, skill and professional behaviours
- Resources
- Emotional Support

The needs were presented to members in the member checking session and, once refined, will be presented in a recommendations section of a manuscript. Literature will be used to evaluate these recommendations. Evidence informed suggestions (from within and beyond this study) of *how* these needs could be met will be stated.

<table>
<tr><th></th><th>System/Service</th><th>Knowledge, skill, professional behaviour</th><th>Resources</th><th>Emotional support</th></tr>
<tr><td colspan="5" align="center">THEME 1: SUBMERGED</td></tr>
<tr><td>1.1 Circle of Poverty</td><td>• Systemic development
• OT resourcing in other sectors (?CS)</td><td>• Population intervention / promotion & prevention</td><td></td><td rowspan="5">• Preparation for CS (knowing what to expect)
• Processing experiences: debriefing / sharing
• Supported reflection</td></tr>
<tr><td>1.2 Strained System**</td><td></td><td>• Managing & strengthening sustainable services
• Navigating myself in a system with a history
• Personal & professional skills (incl. resilience)</td><td></td></tr>
<tr><td>1.3 Contraptions & contractures</td><td>• Improvement in basic management of hand injuries/conditions
• Strengthening human resources (medical & surgical)
• Strengthening referral systems</td><td>• Preventing & treating secondary complications</td><td></td></tr>
<tr><td>1.4 Insurmountable need</td><td></td><td>• Resilience
• Professional skills</td><td></td></tr>
<tr><td>1.5 Connecting through diversity</td><td>• Human resourcing to reduce language discordance
• Interpreters</td><td>• Cultural humility
• Learning additional languages
• Effective use of interpreter
• Responding to traditional healing practices</td><td>• Applications (Apps)</td></tr>
<tr><td colspan="5">1.1 OT Comserve posts in other government sectors especially DoE P5
1.1 Agree- patients were mainly laborers and domestic workers who really need their hands for employment and sustaining their families P8
1.1 agree- many patients were unemployed, no electricity, water or working in informal employment P9
1.2 Agree - government needs to employ more OTs and provide more resources for hand therapy as well as in the health sector in general - Burn out is real P8
1.2 Agree- not getting the correct care
1.2 ** Paraphrase: I attended the Rural health conference and this resonates with conversations around systems and services P6
1.3 Agree - mismanagement of hands patients P8
1.3 Varsity level awareness and lectures to the MDT on role of different MDT members could be an idea P3
1.4 Agree- True being the only OT was tough not having anyone to learn from or ask questions P8
1.5 Agree - all different languages and learning different cultures, adding to the meaning and purpose to a patient’s life P8
1.5 agree- loved this part of my experience P9</td></tr>
</table>

<table>
<tr><th></th><th>System/Service</th><th>Knowledge, skill, professional behaviour</th><th>Resources</th><th>Emotional support</th></tr>
<tr><td colspan="5">THEME 2: STARTING SOMEWHERE</td></tr>
<tr><td>2.1 No idea to some idea</td><td></td><td>• Guidance
• Feedback
• Support for the development of clinical reasoning</td><td></td><td rowspan="5">• Debriefing /processing / sharing experiences

• Preparation for CS</td></tr>
<tr><td>2.2 Finding directions</td><td></td><td>• Guidance
• Feedback
• Support for the development of , knowledge, skill & clinical reasoning (Ax & Rx)
• Accessing appropriate evidence
• Mx acute conditions & injuries
• Mx chronic conditions
• Mx Burnt hand
• Mx fractures
• Mx stroke UL
• Reasoning around DG eligibility</td><td>• Appropriate guidelines</td></tr>
<tr><td>2.3 I know there's a protocol but…</td><td></td><td>• Supporting adherence</td><td>• Appropriate guidelines
• Appropriate home programmes
• Mobile Ax & Rx resources</td></tr>
<tr><td>2.4 Sparse equipping</td><td>• Human resourcing
• Strengthening supervision capacity</td><td>• Strengthening UG Curricula
• Practical UG experiences
• Regular opportunities to develop knowledge & skill
• Strengthening resourcefulness, creativity & innovation</td><td>• Appropriate HT equipment & resources</td></tr>
<tr><td>2.5 Seeing hands as OT</td><td></td><td>• Fostering enjoyable UG hand experiences;
• Strengthening occupational focus in UG HT teaching
• OBHT (Occupation-based hand therapy) opportunities
• Understanding indigenous occupations & drawing them into therapy</td><td>• OBHT resources suitable for diverse caseload
• Activity ideas</td></tr>
<tr><td colspan="5">2.1 Some idea and getting more experience P8
2.2 Agree - lack of diagnosis which means we have to treat symptoms P8
2.3 Agree - patients come so late for therapy and are mismanaged - where to go from there P8</td></tr>
</table>

2.4 Agree - running out of splinting materials and not having the tools that we need to do therapy, have to be creative and use all sorts of items that could work instead P8
2.4 AGREE P9
2.5 agree – loved the positive spin P9
2.5 Agree - OT is hands P9

	System/Service	Knowledge, skill, professional behaviour	Resources	Emotional support
**THEME 3: DYNAMICS OF SURTHRIVAL**				
**3.1 Agentic momentum**		• supporting development of agency<br>• supporting development of lifelong learning practices		
**3.2 Propelled by demand**			• Resources for diverse caseload	
**3.3 Emotional supports**	• Responsive supervision	• Resilience (incl. coping strategies, building/harnessing support networks)		• Preparation<br>• Debriefing /processing / sharing experience
**3.4 Resources**	• Strengthening referral systems		• Appropriate resources	
**3.5 Support for learning**	• ? Appropriate courses	• Strengthening UG experiences<br>• Individual & group reflection<br>• Case discussion	• Appropriate courses	
**3.6 Catalysts**	• Accessible (immediacy & proximity) support & supervision<br>• Access to expertise		? Access to expertise	

- Some kind of mentorship program where you can have access to a experience hand therapist P5
- Community of practice honestly made my comm serv experience P9
- 3.2 Agree - had to go and ask for support from OTs at the hospital P8
- 3.3 Agree -identified the need for OT supervisor and Pets P8
- 3.5 Agree - colleagues help phone for help P8
- 3.6 Agree - real time support P8

Appendix A: Individual vignettes

**P1: Illusionless optmist**<br>Welcome to my corner of the hand therapy world... I work in District hospital in a deep rural town (more of a village really) nestled in the Eastern Cape mountains, close to the foothills of the Drakensburg. Beautiful, but very rural! Resiliently optimist, I embarked on my rural Community Service in a bubble of big dreams. My excitement was soundly grounded by an environment so poorly-resourced, I had no idea how to provide hand therapy. Most of the time hand therapy patients don't recover as service and resource constraints mean that they aren't able to access surgery or rehabilitation. Despite this, I see 20-30 patients with hand injuries or conditions each month about 30% of my caseload. One patient was a young man, Uuka. It was incredible that, through reaching out to experts, I was able to figure out that he had a peri-lunate dislocation – something I would never have been able to work out on my own. It was also really sad though; despite being able to figure out what was wrong, he decided to decline treatment because he was worried about losing his job and would rather have pain than no movement. Surgery would also have been really difficult for him to access. I guess this also highlights the often-perceived futility of working in a setting like this - even when you know what is wrong and know what should be done, generally patients still don't have access or resources to get the help that they need.
**P2: Dedicatedly winging-it**<br>Welcome to my corner of the hand therapy world... I deliver clinic-based services in rural KwaZulu Natal. I treat around 30 patients with hand conditions each month. My first few weeks was the most overwhelming thing I've ever experienced: treating serious hand injuries with no undergraduate hands experience, no OT colleagues, a supervisor 20 km away, and working out of a residential parkhome. How could *I* be expected to treat three-year old Kagabu's burnt hand? This shouldn't be allowed!<br>But I got by. I read and called my supervisor (I probably could have read more). I did enough to get by. At times I dissociated myself from the experience to cope. But it got better. I made splints and pressure garments for Kagabu. I remember a surge of joy as I watched him, hesitatingly at first, but with growing confidence, use his burnt hand to play. He did it! I did it!
**P3: Eager and willing**<br>Welcome to my corner of the hand therapy world...a township clinic situated on the outskirts of the large City of Johannesburg. A small part of my patient load are patients with hand impairments. Some patients have sustained assault injuries and are referred to me when discharged from the City's hospitals. But most of my patients have chronic conditions that affect their hand function – cerebral palsy, CVAs, congenital conditions.<br>With limited knowledge and experience, and very few resources, it's very difficult to feel like I'm actually able to help patients achieve improved function and participation. I'm eager and willing to do everything that I can – but that often feels like it falls very short of the need. The services aren't terrible, but the system isn't great and offering quality OT service feels like what a sunflower might feel growing through mud.
**P4: The Bad Grad**<br>Welcome to my corner of the hand therapy world... a large Hospital in Gauteng. I've rotated through different units in the large OT department treating around 100 hand patients per month when I was on my orthopaedics rotation. I knew nothing leaving uni but thankfully I had a supervisor who was a hands expert and was able to teach and guide me.<br>Despite being a better-resourced hospital, we still spent much of our time dealing with late referral tendon injuries and trying to fix the doctors' mistakes (ie. lack of experience, mismanagement and late referral).
**P5: Tired and Trying**<br>Welcome to my corner of the hand therapy world... a tiny rural hospital in the Eastern Cape, nestled between rondawels, rolling hills and the ocean. Not much around besides goats, cows and a few chickens. I saw about 40 patients with hand injuries each month – close to half of my caseload. Hands has been frustrating, yet rewarding when you get it right – like with 9-year old Andiswa who had a severely burnt dominant hand but was able to return to school.
**P6: Anx-cited outsider**

Welcome to my corner of the hand therapy world... a Rural Regional Hospital in Mpumlalanga. It's a world so different to the one I know. I'm an anxious outsider: feeling out of place, out of my culture, but eager to learn. I was involved in a weekly Hands clinic at the hospital and I saw around 30 hand-injured patients per month. I also did frequent clinic outreach and home visits. Despite a lack of resources and guidance, I learnt a lot and I was able to consolidate my university foundation.<br>My most memorable patient, Thulani, was stabbed in the head with a knife by someone trying to steal his beer; at a time when COVID-19 restrictions made beer scarce.
**P7: The Find-a-way OT**<br>Welcome to my corner of the hand therapy world... A large rural hospital in Limpopo – a very hot part of the world! I see around 30 hand-injured patients each month. Hand therapy is challenging and my clinical reasoning is being put to work. Patients are deeply grateful for any service that I'm able to offer them. But resources are very limited and the support and supervision that I receive is much less than I need
**P8: Solo worker bee**<br>Welcome to my corner of the hand therapy world... I serve urban clinics in the Gauteng Province. I drive from clinic to clinic, carrying the world (my therapy things) with me. I see about 40 patients with hand conditions each month. Many of them have chronic hand problems but don't have formal diagnoses. *Seeing what I can do* summarises my experience.
**P9: The growing therapist**<br>Welcome to my corner of the hand therapy world... here in deep rural Mpumalanga. I work at a district hospital and we service 16 clinics in the area. About 50% of my caseload are patients with hand injuries – many traumatic. I see a lot of victims of abuse and violent crime. Hands was daunting to start with and I lacked confidence. Although I was the therapist with the least experience in my department, I had the most 'hands' knowledge. But, with time, I became more comfortable with hands and enjoyed treating these patients. Seeing patients being able to return to work after a hand injury was very rewarding.

Appendix B: Conceptual Framework

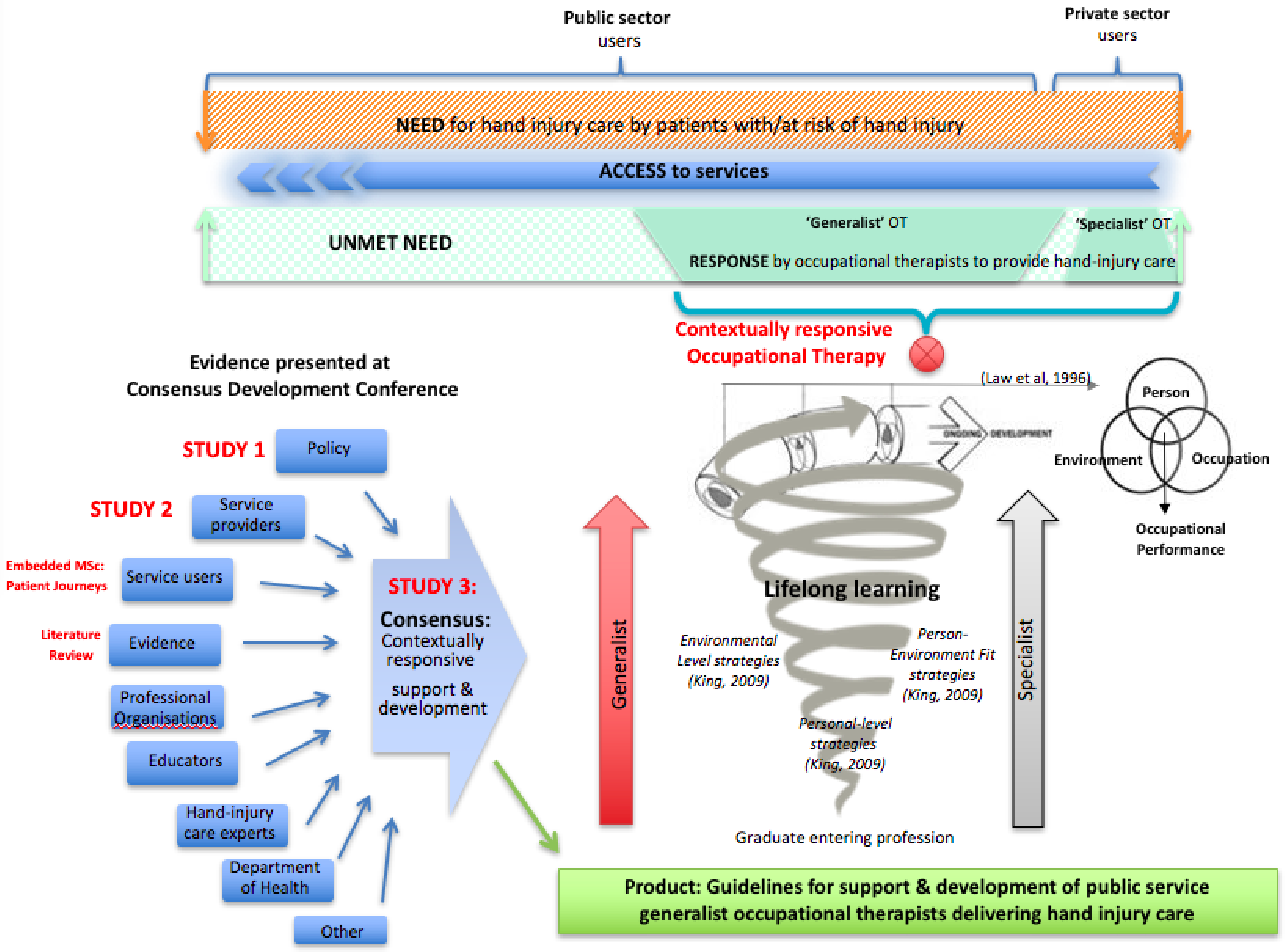

Supplement: Supplementary 3 — Report of analysis process submitted for auditing. [file 5562025.f3.pdf]
